# Basalt geochemistry reveals high frequency of prehistoric tool exchange in low hierarchy Marquesas Islands (Polynesia)

**DOI:** 10.1371/journal.pone.0188207

**Published:** 2017-12-27

**Authors:** Andrew McAlister, Melinda S. Allen

**Affiliations:** 1 Anthropology, School of Social Sciences, University of Auckland, Auckland, New Zealand; 2 Anthropology, School of Social Sciences, Te Pūnaha Matatini, University of Auckland, Auckland, New Zealand; University of Hawaii at Manoa, UNITED STATES

## Abstract

Exchange activities, formal or otherwise, serve a variety of purposes and were prominent in many Pacific Island societies, both during island settlement and in late prehistory. Recent Polynesian studies highlight the role of exchange in the region’s most hierarchical polities where it contributed to wealth economies, emergent leadership, and status rivalry in late prehistory. Building on this research, we hypothesized that exchange in low hierarchy chiefdoms (kin-based polities where there are distinctions between commoners and elites but ranking within the latter is lacking, weak, or ephemeral) would differ in frequency and function from that associated with strongly hierarchical polities. We address this hypothesis through geochemical, morphological, and distributional analyses of stone tools on Nuku Hiva, Marquesas Islands. Non-destructive Energy-Dispersive X-ray Fluorescence (EDXRF) and destructive Wavelength-Dispersive X-ray Fluorescence (WDXRF) analyses of 278 complete and broken tools (adzes, chisels, preforms) from four valleys identify use of stone from at least seven sources on three islands: five on Nuku Hiva and one each on Eiao and Ua Pou. A functional analysis demonstrates that no tool form is limited to a particular source, while inter-valley distributions reveal that the proportions of non-local or extra-valley tools (43 to 94%, mean = 77%) approximate or exceed results from other archipelagoes, including those from elite and ritual sites of Polynesian archaic states. Intra-valley patterns also are unexpected, with non-local stone tools being recovered from both elite and commoner residential areas in near-equal proportions. Our findings unambiguously demonstrate the importance of exchange in late prehistoric Marquesan society, at varied social and geographic scales. We propose the observed patterns are the result of elites using non-local tools as political currency, aimed at reinforcing status, cementing client-patron relations, and building extra-valley alliances, consistent with prestige societies elsewhere and early historic accounts from the Marquesan Islands.

## Introduction

Human exchange systems serve a variety of functions, including distributing scarce resources, promoting social cohesion, and mitigating environmental perturbations [1–5]. Exchange also can be an effective elite strategy for enhancing status, aiding centralization, and underwriting hegemonic expansion [6–11]. Both utilitarian and prestige items may be involved and, notably, the social interaction itself may be as important as the mobilized goods [12]. Exchange is a prominent feature of many indigenous Pacific Island societies, and has been the subject of considerable ethnographic and archaeological research [6,9,13–16]. In Polynesia, geochemical analysis of durable stone tools has proven an effective way to track patterns of exchange and interaction [17–19]. These studies reveal remarkable long-distance voyages between archipelagos, coincident with and following from early East Polynesian settlement [10,20–23]. They also identify a prominent role for imported stone goods in the development of wealth economies, increasing socio-political complexity, and the appearance of archaic states in late prehistory [7,8,24,25]. It is these later processes that are of interest here, and questions of if, and how, exchange figured in the socio-political landscape of Polynesia’s low hierarchy chiefdoms. These are defined here as kin-based polities where there are distinctions between commoners and elites, but ranking within the latter is lacking, weak, or ephemeral.

The potential contribution of exotic stone tools (particularly adzes) to the economic wealth of Polynesian chiefdoms has long been considered, but only recently demonstrated, largely through basalt sourcing studies. Kirch and colleagues [24], for example, used X-ray Fluorescence (XRF) analysis to show the importance of non-local (extra-district and extra-island) materials (adzes and flakes) in the stratified and centralized polities of Kahikinui, Hawai‘i, where they figure prominently in tool assemblages from elite residences and ritual sites (temples and shrines). Their research suggests that access and distribution of such resources were controlled by elites, specifically chiefs, subchiefs, land managers, and priests. Extra-island adzes and flakes also are well represented in elite activity areas of the late prehistoric Tongan State, where they represent more than half of the tools and flakes recovered from royal tombs and central places [7]. Along with staple goods, imported stone artifacts were redistributed through regular ceremonial events and were “an important source of capital for Tongan elites to maintain and fund a centralized political system dependent on long-distance canoe voyaging” [7]. In contrast, imported adzes and flakes were comparatively uncommon in pre-state Tongan contexts. In the stratified but less hierarchical chiefdoms of Mo‘orea (Society Islands) non-local (extra-island) tool sources were moderately well represented, comprising around a third of the overall lithic assemblages but, as in the Hawaiian case, were concentrated in ritual and specialized contexts [8]. These studies demonstrate the important place of stone tool exchange in some of Polynesia’s most hierarchical societies and their relative unimportance in low hierarchy contexts (e.g., pre-state Tonga). The Marquesas Islands provide an opportunity to examine the role of exchange in Polynesia’s less stratified and weakly centralized polities, specifically those intermediate between simple strictly kin-based chiefdoms and late prehistoric archaic states, such as those of Hawai‘i and Tonga.

Our analysis examines 278 whole or nearly whole stone tools (adzes, chisels, and preforms), as opposed to flake debitage, from late prehistoric sites spread across four Marquesan valleys on Nuku Hiva Island. Our initial expectation was that the accumulation and distribution of costly non-local (extra-valley) goods would have been advantageous in a society where prestige rivalry was intense, but also challenging given modest levels of centralization and the high costs of acquisition (recruitment and deployment of labor, craft specialists, and transport). We identify several stone sources in the archaeological assemblages, of local, regional, and extra-island origin, through a combination of non-destructive Energy-Dispersive X-ray Fluorescence (EDXRF) and destructive Wavelength-Dispersive X-ray Fluorescence (WDXRF) techniques. We also evaluate functional tool types in relation to raw material source and show that no tool form was limited to a particular raw material, suggesting that exchange was largely motivated by social rather than utilitarian factors. Finally, to understand the underlying social drivers of exchange, we examine the distribution of raw material groups at three geographic scales (inter-district, inter-valley, and intra-valley). Our analysis demonstrates that stone tool exchange was quite important in late prehistoric Marquesan society, but differed from patterns observed in more strongly hierarchical and centralized polities elsewhere, and in unexpected ways.

## Background to the Marquesas Islands

### Environment

**Fig 1:**
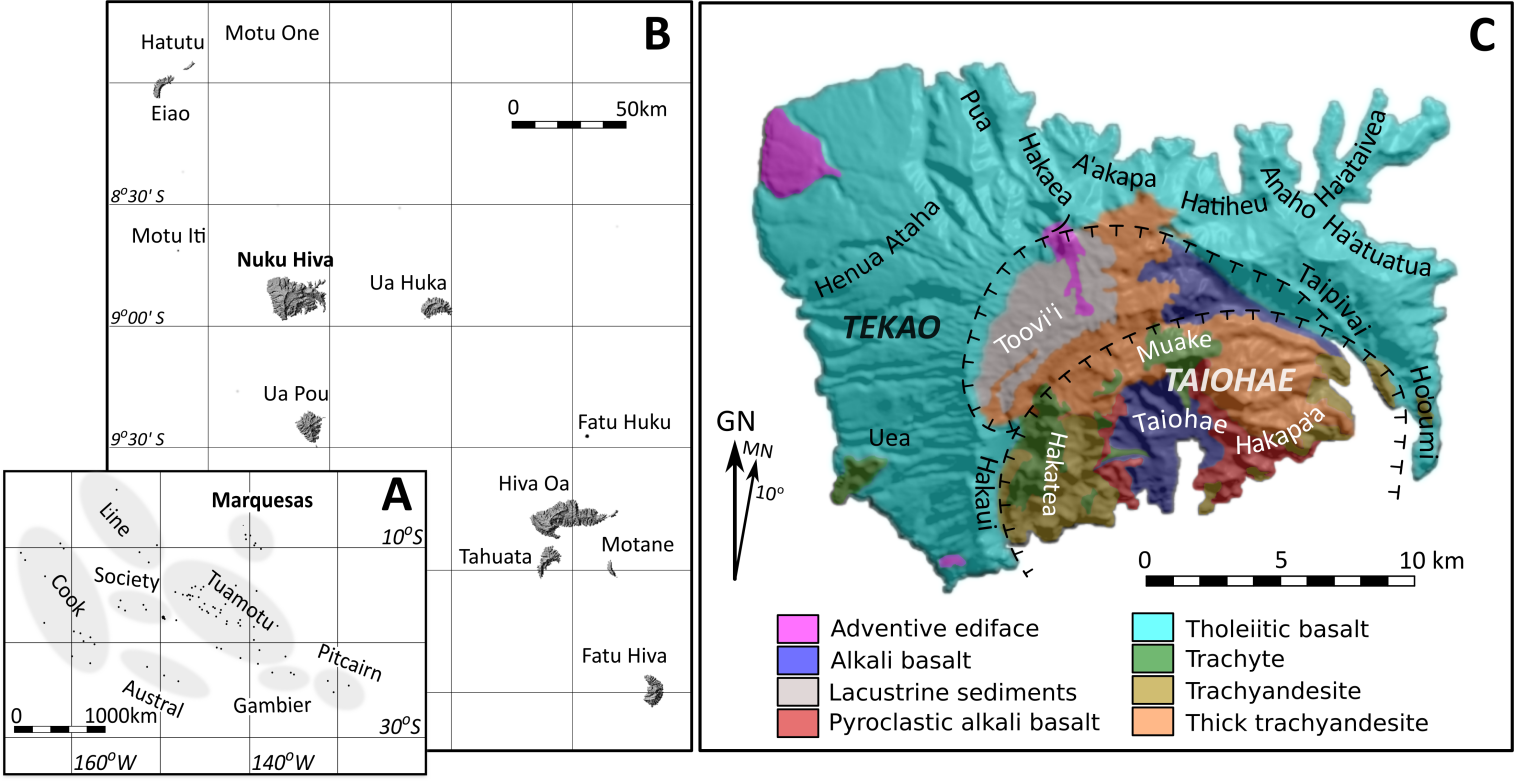
Study area. A. Central East Polynesia; B. Marquesas Islands; C. Nuku Hiva, major valleys and volcanic zones (after [26]).

The Marquesas Archipelago consists of eight main islands, thought to have formed through hotspot processes (Fig 1), with ages decreasing from the northwest (Eiao, 5.52 Ma) to southeast (Fatu Hiva, 1.1 Ma) [26]. Most of the islands are composed of partially collapsed outer shield volcanoes derived from depleted (peridotitic) plumes, which were succeeded by smaller enriched (pyroxenitic) post-shield volcanoes. Although formed by similar processes, the underlying mantle is highly heterogeneous, resulting in considerable variation in rock texture and chemistry both within and between islands. For instance, Ua Pou has an exceptionally high proportion of phonolites [27], while Eiao possesses deposits of unusually fine-grained aphyric tholeiites and alkali basalts [28,29]. On Nuku Hiva (339 km^2^), the outer Tekao shield volcano covers much of the island and is largely composed of tholeiitic basalts (Fig 1). The more recent Taiohae post-shield volcano, centered on Taiohae Bay, derives from an enriched mantle and has a more complex geology [30].

These volcanic processes, in combination with high rainfall and a tropical climate, have produced a rugged topography and often deep, narrow valleys, which limit human settlement and challenge inter-valley mobility. With restricted coastal flats and few alluvial floodplains, traditional large-scale irrigated agriculture was not possible and over time Marquesan communities came to rely on arboriculture [31], with breadfruit being the primary carbohydrate at Western Contact [32,33]. Although the climate is mild, precipitation extremes regularly posed threats to human livelihood in the past. Frequent and often extended droughts devastated food and water resources, while extreme rainfall caused flooding, significant run-off, and sometimes massive erosion. These climatic perturbations had some periodicity, but their amplitude, duration, and geographic effects were variable and unpredictable [34].

### Cultural background

Early historical accounts give insights into the socio-political character of traditional Marquesan society. While these records should not be uncritically applied to the past, they provide a baseline against which the late prehistoric (ca. post-1650 AD) archaeological assemblages examined here can be compared. The most valuable Contact-period records derive from the earliest Western residents of the late 18^th^ to early 19^th^ centuries [32,33,35,36]. These accounts suggest two pathways to leadership, one through genealogical links and a second via demonstrated abilities and accomplishments. In the main, two social classes were recognized, commoner and elite—the latter comprised of hereditary chiefs, warriors, and priests. Individuals from these three elite groups vied for political, economic and, to some extent, religious authority, while commoners were linked to elite households in labor-tribute relations [37]. Within the elite class, social rankings were apparent but complex, fluid, and not easily categorized by outsiders. In his detailed ethnohistorical analysis, Thomas [36] suggests that early Contact-period Marquesan society was “structured by varieties of difference and inequality unconnected with the sanctity of chiefs and chiefly lines” (pg. 87). Inferior leaders could be replaced, and accomplished or generous individuals could rise to power, occasionally even when genealogical links were lacking. Thus warriors, priests, and occasionally propertied non-elites, actively competed with hereditary chiefs for leadership positions and the control of non-kin labor and production [34–37]. The latter included labor for communal enterprises such as temple construction and breadfruit harvesting, and specialized craft production, such as wood and stone carvings [32,33,35,38]. Other distinctive socio-political features, such as institutionalized polyandry and privatization of certain resources, acted to disperse power and decision-making [34]. Early accounts suggest that political units rarely exceeded single valleys and while tribes were occasionally united into larger polities, such alliances were typically ephemeral. Competition for leadership also occurred between tribal groups, but in these cases was more resource-oriented, and often involved competitive feasting, sacrificial raids, destruction of food resources and, less frequently, large-scale battles. Some inter-polity competition apparently had deep historical roots, as for example on Nuku Hiva Island where the origin of two loosely organized supra-tribes, the western Tei‘i and the eastern Taipi, was attributed to eponymous sibling rivalry [36].

Notably, Contact-period Marquesan society was the outgrowth of some seven centuries of adaptation, population growth, and internal socio-political dynamics. Archaeological evidence places initial Marquesan settlement in the 11^th^ to 13^th^ centuries AD if not earlier [39–41]. Suggs [42] and others document the growth, spread, and consolidation of communities over the ensuing centuries. From the mid-17^th^ century, Marquesan landscapes were dominated by dry stone masonry house foundations of variable size and complexity [43]. Less common and more poorly dated are the large, spectacular ceremonial structures (*tohua*), which were the focus of community feasting and song and dance performances [41,44,45]. *Tohua* in particular point to powerful elites who could command and organize large labor pools, and direct them to challenging feats of engineering [44,45]. Other large structures of variable size, complexity, and form, and often in distinctive locations, were likely religious in function (*me‘ae*) and the purview of powerful and sometimes much feared priests [44,46]. Overall, while the subtleties of Marquesan social rankings alluded to in ethnohistorical accounts may not be archaeologically tractable, elite activities in general are clearly recognizable through the materialization of their economic, political, and religious activities.

### Stone tool sources

The most important and extensive Marquesan stone tool raw material sources are found on the 43.8 km^2^ island of Eiao at the northern end of the archipelago (Fig 1) [38,47,48]. Eiao’s rugged coastline makes access difficult, even with modern watercraft, and lives were sometimes lost in making this 100-km journey [32]. Limited fresh water resources constrained permanent occupation, at least in the historic period and probably in the more distant past. Island use is indirectly dated from the 12^th^ century AD by Eiao tools identified in sites located elsewhere in the archipelago [41,49]. Several quarries and adze workshops have been recorded on the island, along with house foundations (*paepae*), and ceremonial and religious structures that are consistent with mid- to late-prehistoric architectural styles [44,47,50,51]. Although little is known about the organization and control of Eiao tool production, there are indications that the final finishing stages of adze production were typically carried out off-island [44,47,50]. The possibility that Nuku Hiva functioned as a distribution center for Eiao products also has been raised, largely on the basis of oral traditions [38].

Eiao rock is visually distinguished by its fine texture and dark color. Finished Eiao stone adzes have been recovered from both northern and southern Marquesan sites, occurring throughout the prehistoric sequence [41,49,52,53]. Indeed, the geographic distribution of Eiao stone tools exceeds that of any other East Polynesian source, extending east to the Cook Islands, north to the Line Islands, and south to Mangareva, where it is often found in early contexts [21–23,39,54,55]; these extra-archipelago findings indicate that the island’s stone resources were recognized and valued from the earliest phase of East Polynesian settlement. On Tahuata Island in the southern Marquesas, Rolett [41] identified Eiao stone in early basal contexts, but found that after 1450 AD its importation declined in relation to more proximate non-local sources. While Eiao stone was undoubtedly sought-after, materials of sufficient quality for tool manufacture were widely available on Nuku Hiva. Three quarries are known from the northeast coast, one each in Ha‘ataivea, Anaho, and Hatiheu Valleys, and another is located in the western Henua Ataha region [42,49,52]. These sources are generally moderate-sized dyke-stone exposures but numerous other minor sources are known from several valleys.

## Materials and methods

### Sample selection

Artifacts (n = 278) from four valleys on the northern coast of Nuku Hiva (Pua, Hakaea, Hatiheu, and Anaho) were analyzed. Complete, reworked, and broken adzes, chisels, and preforms (as opposed to flakes) were selected to provide robust estimates of source proportions, as adze manufacture and rejuvenation both produce numerous flakes and can bias source abundance estimates [56]. Our study assemblages derive from systematic surface collections in and around late prehistoric-early historic domestic structures and, in a limited number of cases (n = 7), from post-1650 AD occupation layers. Collections from Anaho Valley, the site of a long-term research program [43], are complemented by more targeted collections in the valleys of Hatiheu, Pua, and Hakaea [49,52,57]. Research permits for our study were provided by the French Polynesian Government Délégation à la Recherche and the French High Commission Délégation Régionale à la Recherche et à la technologie.

In addition to artifact assemblages, our dataset includes reference samples collected from sources on Nuku Hiva, Eiao, and Hiva Oa. This reference set totals 497 samples, 263 analyzed for this study and an additional 234 from other studies. The majority of the reference specimens consist of unworked raw materials but in some cases manufacturing debitage also was included, as noted below. These samples were placed into eight source groups based on geochemical and geographical similarities, as described below.

### Northeast Nuku Hiva (n = 158)

This group comprises reference samples collected from the four adjacent, northeast valleys of Nuku Hiva (Ha‘atuatua, Ha‘ataivea, Anaho, and Hatiheu). Three distinct quarries are included in this geochemical group, one in Ha‘ataivea [42], one in Anaho [49], and one on the east coast of Hatiheu [52]. Additionally, several dykestone outcrops, some in proximity to lithic scatters that might represent either small-scale quarrying or opportunistic exploitation of suitably fine-grained stone, were sampled [49]. The samples from all of these sources are located within a two km radius and possess a similar geochemistry that cannot be separated by geographical location.

### Atikea, Nuku Hiva (n = 33)

Samples of fine-grained basalt collected from the interior of Hatiheu Valley have a geochemistry distinct from those of the east coast of Hatiheu Valley and also from other northeastern Nuku Hiva valleys noted above, and so were placed in a separate geochemical group. These specimens were not associated with a specific adze production locality but were sampled from outcrops, boulders, and large cobbles. To avoid confusion with the coastal source in the same valley, this geochemical group was designated Atikea, after the traditional name of the associated land unit and tribe [58,59].

### Northwest Nuku Hiva (n = 31)

This geochemical group includes specimens struck from outcrops and boulders of fine-grained basalt in the western valleys of Hakaea and Pua. No direct evidence of stone quarrying has been identified in these valleys, although some of the specimens were collected from un-modified boulders adjacent to lithic scatters [49]. The samples are, however, of a quality suitable for adze manufacture. Again, there is considerable geochemical overlap in the samples from these valleys, so they were grouped together for analytical purposes.

### Henua Ataha, Nuku Hiva (n = 12)

Henua Ataha, located in the arid Terre Déserte region of central, western Nuku Hiva, consists of two large dykestone exposures, both associated with large amounts of flake debris. Identified by other researchers (P. Ottino, J-F. Butaud, and T. Maric), a full account has yet to be published. Twelve debitage flakes were collected from this site and given to the authors by Dr. Tamara Maric of the Service de la Culture et du Patrimoine, Puna‘auia, French Polynesia. This material is similar to samples from other northern locations in appearance and generally in its geochemical composition, but possesses distinctive concentrations of nickel (Ni) and chromium (Cr).

### Taiohae volcanic region, Nuku Hiva (n = 23)

Fine-grained specimens were collected from several locations in the southern region of Nuku Hiva, mainly from outcrops and riverbed boulders in the valleys of Taiohae and Taipivai. These materials derive from the Taiohae inner-shield volcanic stage of Nuku Hiva’s formation. They are geochemically similar to one another but distinct from materials from the north of the island, which were formed during the initial Tekao volcanic stage [26]. Although not systematically surveyed, impressionistically stone from the Taiohae Caldera is more coarse-grained than that from the northern Nuku Hiva valleys.

### Eiao Island Group I (n = 211)

The Eiao Island specimens included in our reference set derive from previously published analyses. They were collected from various locations throughout Eiao Island by Charleux and colleagues [47], and include samples from natural rock outcrops as well as artifacts (flakes, preforms, and rejects). Stone from this source is geochemically homogenous, very fine-grained and, when flaked or polished, is black in color. As discussed above, adzes from this Eiao source were distributed throughout the Marquesas Islands in prehistory, and in small numbers to other East Polynesian archipelagos.

### Eiao Island Group II (n = 23)

A second geochemically distinct group of coarser and often reddish colored rock from Eiao also was reported by Charleux and colleagues. Evidently, this source was not as commonly used as Eiao Island Group I; to date only one adze made from this material, collected on Hiva Oa Island, has been identified [47].

### Hiva Oa Island (n = 6)

Geochemical data for adze-stone sources on Hiva Oa Island is lacking. We include six samples taken from different portions of two boulders in Atuona Valley by Allen in 1995. They are not associated with a traditional quarry but are fine-grained material. Only one other sample from Hiva Oa has previously been reported [17]. This is a flake from the Hanatukua Shelter (Sample AN-46; MH-21-90). Its major element chemistry compares well with our Hiva Oa samples, but unfortunately it was not analyzed for trace elements so it could not be included in this analysis.

In any provenance study it is necessary to first define a “provenance environment”—an appropriate geographical analytic scale informed by theoretical and practical considerations [60]. Previous studies have shown that Marquesan adze sources can be distinguished geochemically from other known Polynesian sources [17,21,49,54] and, to date, no extra-archipelago stone has been identified in archaeological contexts within the Marquesas Islands [41,47,49,53]. On this basis, we limit our provenance environment to the Marquesas Islands. As is the case for most regions, geochemical data for all potential adze sources in the Marquesas Islands are incomplete. To gain a broad understanding of geochemical trends in the archipelago, our initial analysis draws on additional data compiled in the GEOROC online database (n = 408), a repository for geological research [61].

### Geochemical analysis

All archaeological and reference specimens were analyzed non-destructively with a Bruker Tracer III-SD EDXRF analyzer. Each specimen was analyzed three times on different surface areas and the results were averaged. A sample of the specimens (n = 67) was selected from the four major identified geochemical groups (Eiao Group I, Northeast NH, Henua Ataha NH, Northwest NH) and subjected to destructive but more accurate WDXRF analysis. At the conclusion of the geochemical investigation, the WDXRF and EDXRF results were compared. As expected, the EDXRF data show more within-group variability, reflecting the lower precision of that technology (see Table B in S1 Appendix). However, the similarity of group means suggests no systematic bias between the two methods. Analytic procedures and calibration details for both techniques are reported in S1 Appendix and the calibrated results in S1 Dataset.

## Results

**Fig 2:**
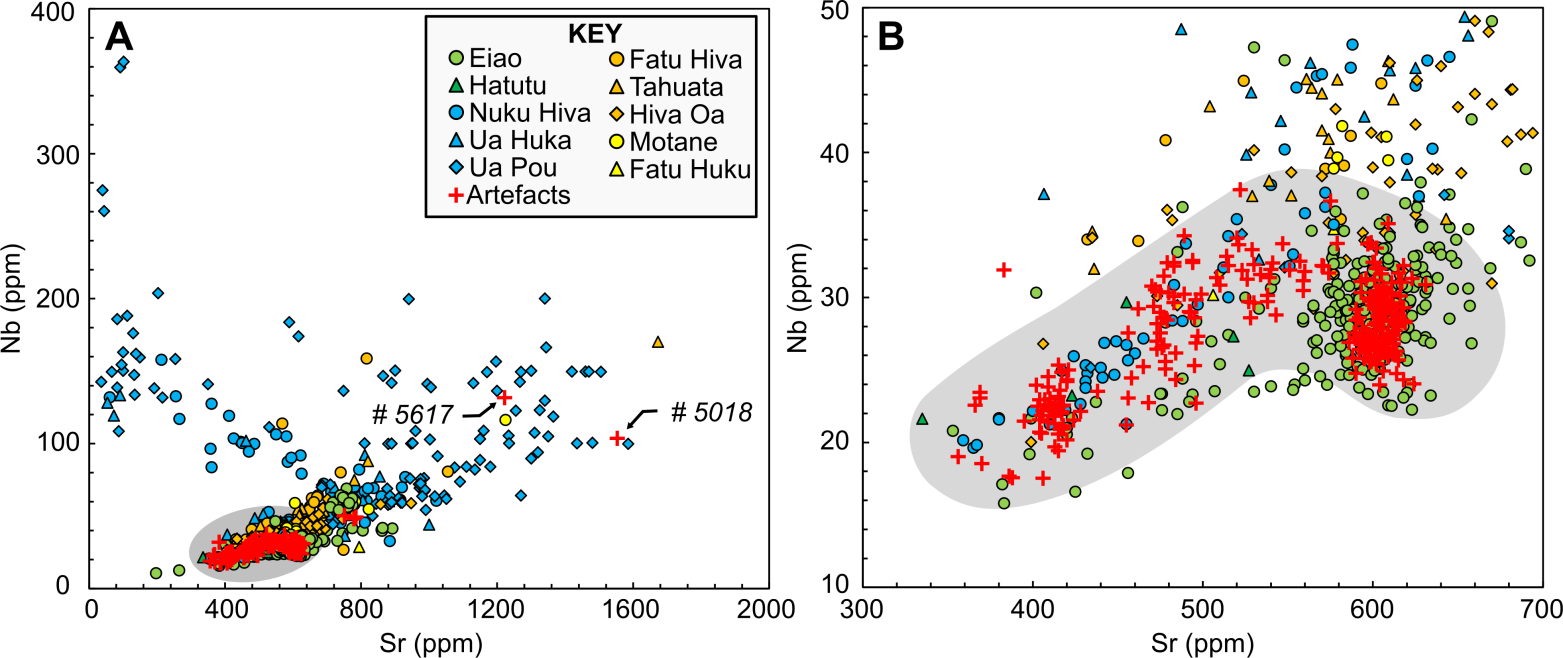
Scatterplot of Sr versus Nb for Marquesan rock samples by island. The reference dataset combines the specimens analyzed for this study with those in the GEOROC database (n = 905). Fig 2A shows the entire range of concentrations; Fig 2B shows the region including the majority of the artifacts. The main artifact cluster is enclosed in gray.

Initially, we used bivariate scatterplots, including both the geological and archaeological reference data, to broadly identify potential sources for the artifacts. Two specimens (Samples 5018 and 5617) with high concentrations of strontium (Sr) and niobium (Nb) are distinct from the main artifact group and cluster with phonolites and basanites from Ua Pou Island, approximately 30 km to the south of Nuku Hiva (Fig 2A). While it is preferable to match artifacts to specific sources that are known to have been exploited, at present no archaeological data for fine-grained rock sources on Ua Pou are available. Given that these two artifacts possess concentrations of Sr that are not found on Nuku Hiva, they are probably imports. As Ua Pou is the closest geochemical match, we tentatively assign these two specimens to that island. The remainder of the artifacts cluster together in a restricted region of the scatterplot that is dominated by reference specimens from Nuku Hiva and Eiao, suggesting the artifact sources are located on these islands (Fig 2B).

One potential issue with using geological data for archaeological studies is that the range of rocks studied by geologists do not necessarily possess physical properties that are suitable for the manufacture of edged tools, such as adzes or chisels. For example, many of the specimens included in the GEOROC database are described as having porphyritic or vesicular textures. On this basis, and because we have sizable reference collections of fine-grained stone for Eiao and Nuku Hiva sources, we limit the reference data for the remainder of our analysis to the eight fine-grained source groups reported above.

**Fig 3:**
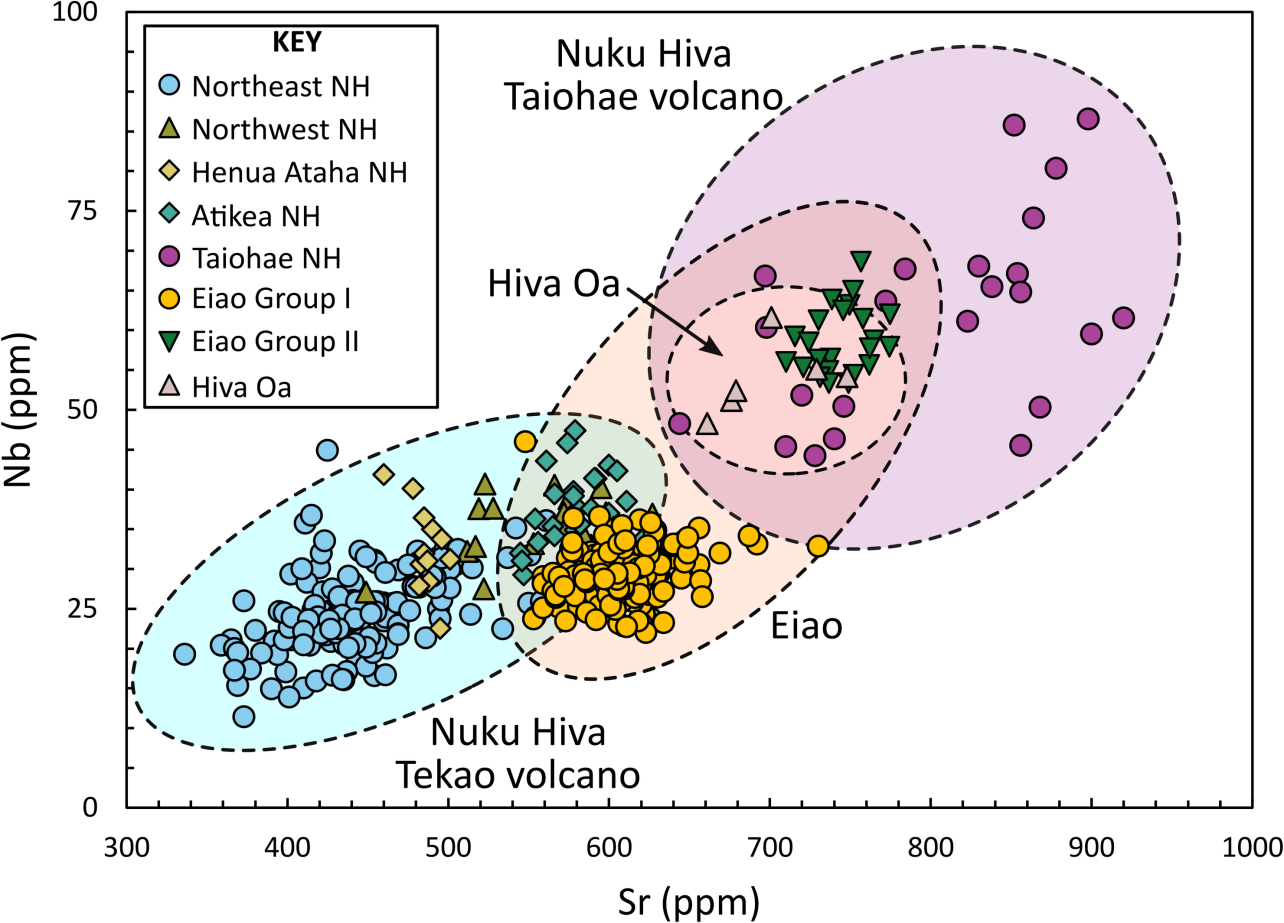
Bivariate plot of Sr against Nb for the fine-grained reference samples.

In previous studies of Polynesian adze chemistry, simple bivariate scatterplots of trace element concentrations or ratios were often sufficient to discriminate known basalt sources [17,20,41,53,62]. However, these studies typically included only small numbers of reference samples and considered relatively few potential sources. In recent years, much larger datasets for many Polynesian adze sources have been reported [8,10,47,63,64]. This has aided our understanding of inter- and intra-source variability, but at the same time has made reliable source discrimination more complex, particularly within limited geographical scales [65,66]. The geochemical reference groups included in this study are all from the Marquesas Archipelago, and five are from the same island. While scatterplots of paired trace elements can show volcanic trends, there is too much overlap to discriminate among individual source areas (Fig 3). Consequently, we employ two supervised multivariate classification techniques to separate geochemical reference groups and assign provenances to the artifacts: Classification Tree Analysis (CT) and Discriminant Function Analysis (DFA).

### Classification tree results

**Fig 4:**
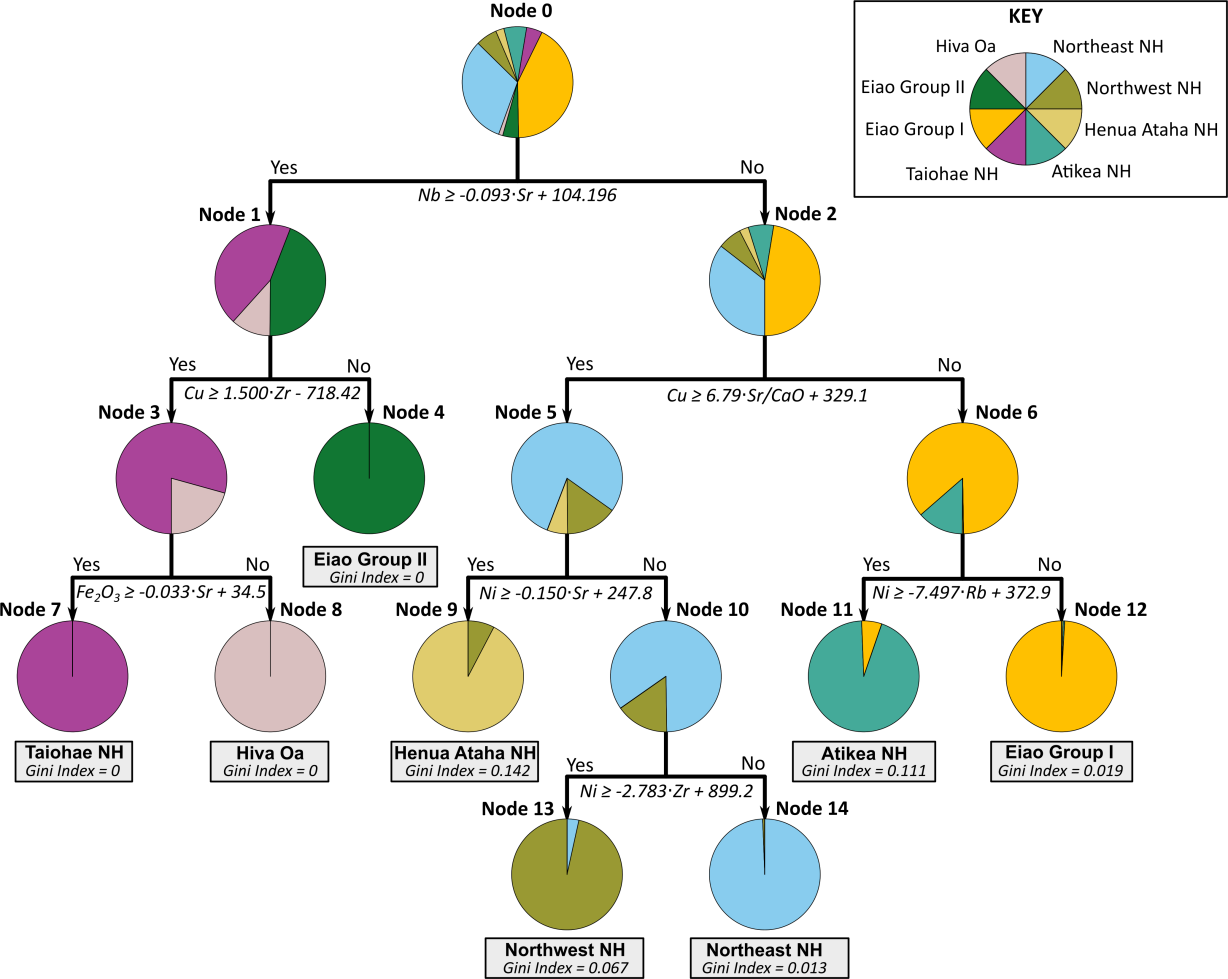
Classification tree for the reference specimens. Terminal nodes are identified in gray.

CT analysis operates by recursively partitioning predictor variables to generate a sequential set of decision rules [67]. Although several software applications of CT analysis with automatic variable selection are available [68,69], they tend to be relatively simple, allowing only one variable or rudimentary combinations of two variables (i.e., sums and differences) to be selected for each partition. While applications such as these can produce satisfactory results [66], trends in geochemical data are better understood when the relationships between two or more elements are examined. To determine partitioning boundaries, we employ *ksvm*, a Support Vector Machine (SVM) learning software package for R [70]. SVM learning encompasses a family of non-parametric maximum-margin classifiers that include complex non-linear algorithms (e.g., polynomial and radial basis function) [71]. However, we found simple linear decision boundaries, in the form *y* = *α x*+*β*, were sufficient to separate the geochemical groups in this dataset. Overall, 490 of the 497 reference samples (98.6%) were classified correctly. The resulting tree structure is shown below (Fig 4, Table 1), along with graphical displays of the decision boundaries (S1 Fig).

**Table 1:** Node membership for classification tree analysis. Modal sources are underlined; terminal nodes are shaded.

	Node														
Source	0	1	2	3	4	5	6	7	8	9	10	11	12	13	14
Northeast NH	158		158			158					158			1	157
Northwest NH	31		31			30	1			1	29		1	28	1
Henua Ataha NH	12		12			12				12					
Atikea NH	33		33				33					32	1		
Eiao Group I	211		211				211					2	209		
Eiao Group II	23	23			23										
Taiohae NH	23	23		23				23							
Hiva Oa	6	6		6					6						
**Artifacts**	**276**	**3**	**273**	**3**		**132**	**141**	**3**		**26**	**106**	**2**	**139**	**11**	**95**

### Discriminant function analysis results

**Fig 5:**
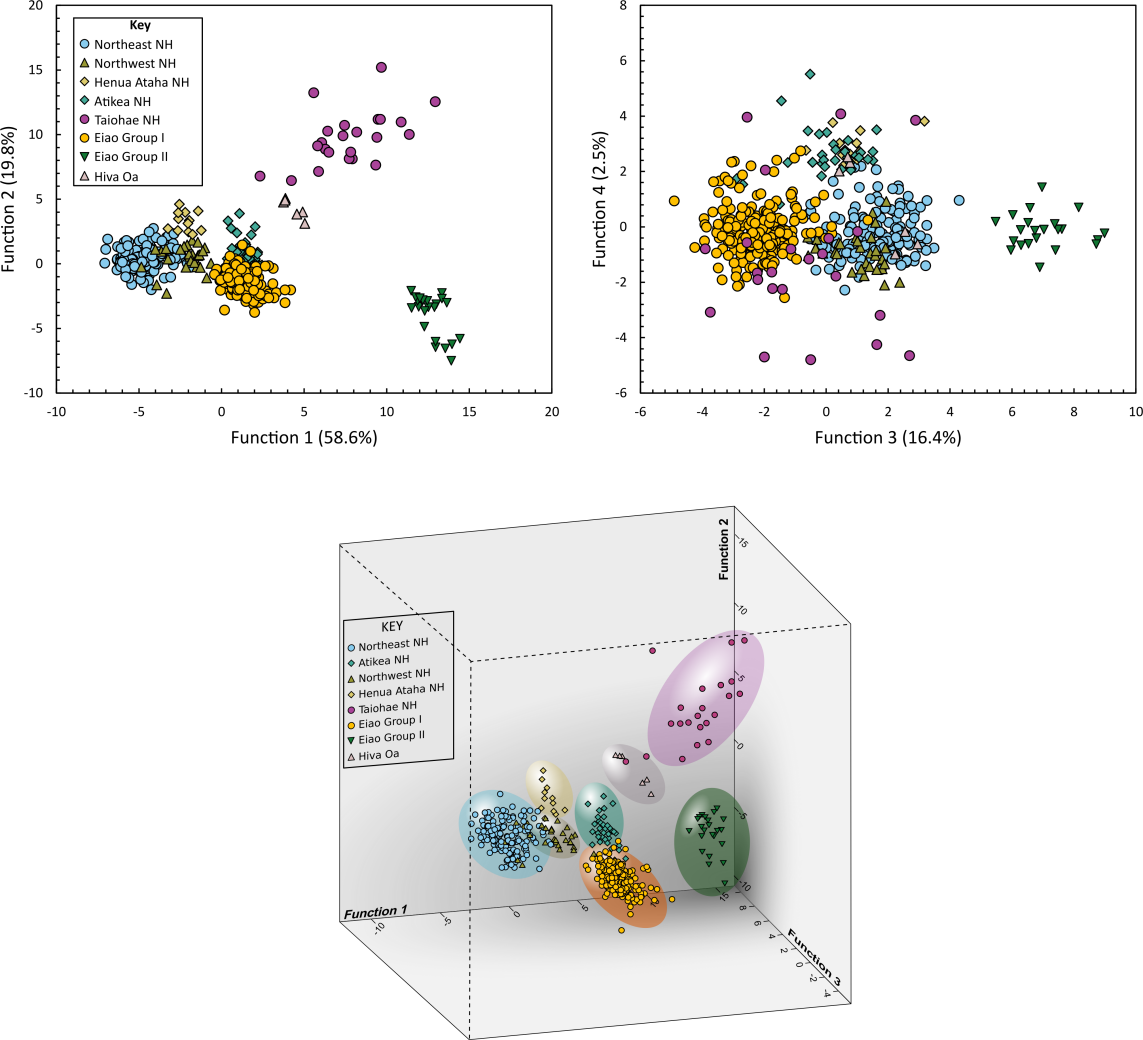
Examples of 2D and 3D projections for the initial discriminant function analysis.

Discriminant Function Analysis is a technique commonly used in archaeological research [72–74]. In contrast to the sequential approach of CT analysis, DFA combines input variables into composite discriminant functions and processes them simultaneously. Initially, we ran a DFA analysis including all eight potential sources using the SPSS (ver. 20) package. This correctly predicted 96.6% of the reference set but the seven resulting discriminant functions are challenging to evaluate visually. For example, the 1^st^ and 2^nd^ functions show a clear separation of the Hiva Oa, Eiao Group II, and South Nuku Hiva sources but there is considerable overlap among the other groups (Fig 5). Similarly, the 3rd and 4th functions separate the Atikea and Eiao Group I samples, but this is obscured by the other sources. Three-dimensional scatterplots show more of the structure but it is difficult to find an ideal rotation that shows maximum separation of all groups and, in this case, only three of the seven functions can be displayed simultaneously (Fig 5).

One solution is to adopt an iterative or “nested” approach to discrimination, in which sources with similar compositions are initially combined and then each of these combinations is analyzed separately [73,75]. In this way, maximum separation can be achieved between the most dissimilar combinations of groups, and then those combinations can be examined. Here, we use a two-stage discriminant analysis—first the entire sample is divided into three similar groups (A, B, and C), then each group is analyzed in turn.

**Fig 6:**
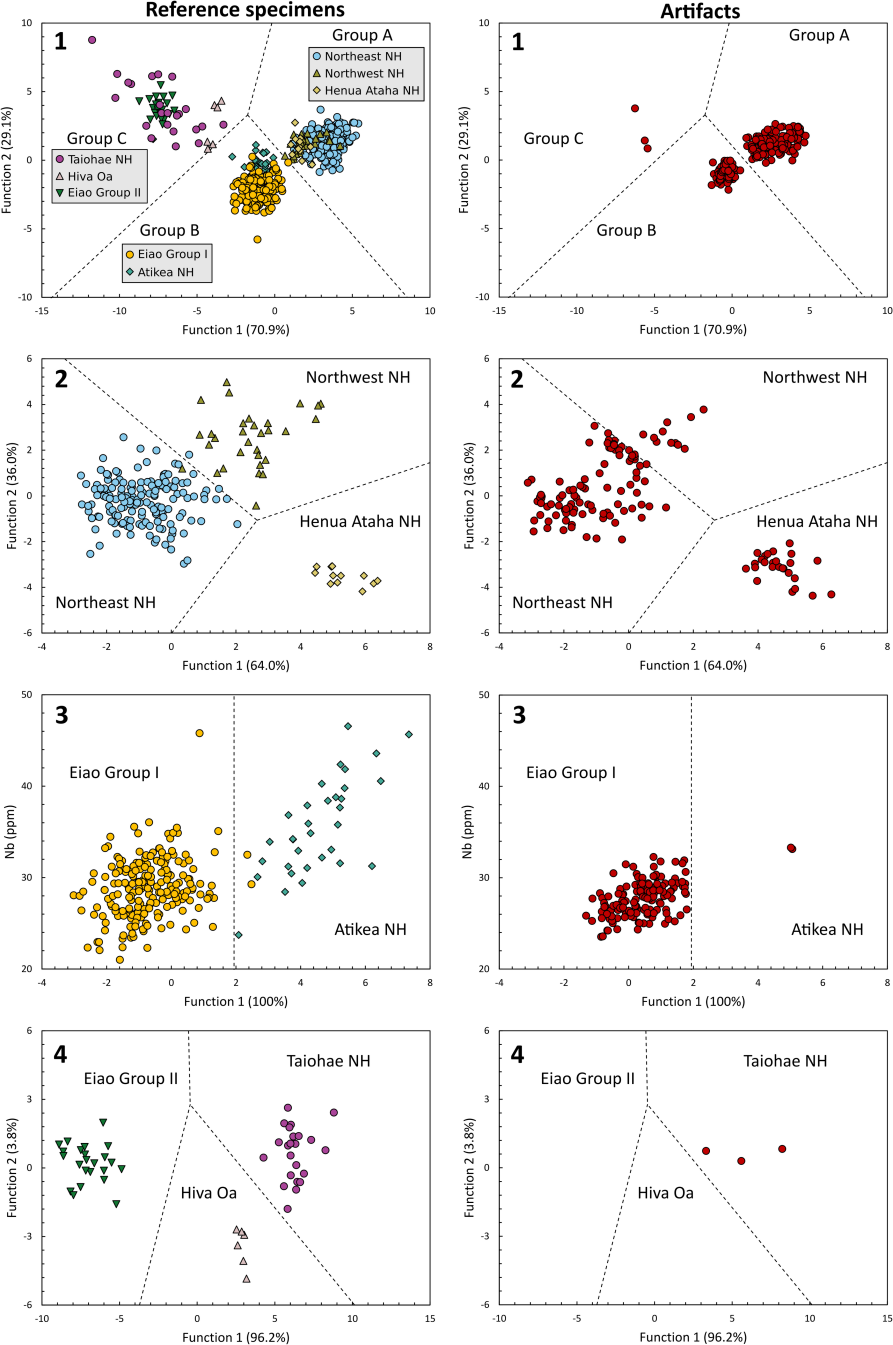
Scatterplots of the discriminant functions. 1. division into three groups; 2. separation of Group A; 3. separation of Group B; 4. separation of Group C. Classification boundaries are shown as dashed lines.

Using the initial analysis as a guide, the three source groups from the north coast of Nuku Hiva (and associated with the Tekao shield volcano) all share a similar geochemistry and are combined as Group A. Group B combines the Eiao Group I and Atikea samples, and the third group (Group C) includes the remaining three sources—Hiva Oa, Eiao Group II, and Taiohae. This approach produced a higher rate of correct classifications (99.2%) and also allowed better visual appraisal of the group separation (Fig 6, S1 Table). Group B included two sources (Eiao Group I and Atikea), and thus produced only one discriminant function. This is plotted against a ‘dummy’ variable (Nb) to allow for bivariate display.

Overall, both methods performed well. Each correctly classified over 98% of the reference specimens, and all artifacts were assigned to the same geochemical sources by both analyses. Details of the results for the two methods are reported in Table 2.

**Table 2:** Comparison of the results for the discriminant function and classification tree analyses.

	Predicted Source								
	Northeast NH	Northwest NH	Henua Ataha NH	Atikea NH	Eiao Group I	Eiao Group II	Taiohae NH	Hiva Oa	Total
**Discriminant Analysis**^**a**^									
**Actual Source**									
Northeast NH	157	1							158
Northwest NH	1	30							31
Henua Ataha NH			12						12
Atikea NH				33					33
Eiao Group I				2	209				211
Eiao Group II						23			23
Taiohae NH							23		23
Hiva Oa								6	6
**Artifacts**^**c**^	**95**	**11**	**26**	**2**	**139**		**3**		**276**
									
**Classification Tree**^**b**^									
**Actual Source**									
Northeast NH	157	1							158
Northwest NH	1	28	1		1				31
Henua Ataha NH			12						12
Atikea NH				32	1				33
Eiao Group I				2	209				211
Eiao Group II						23			23
Taiohae NH							23		23
Hiva Oa								6	6
**Artifacts**^**c**^	**95**	**11**	**26**	**2**	**139**		**3**		**276**

^a^ 99.2% of grouped cases correctly classified.

^b^ 98.6% of grouped cases correctly classified.

^c^ Artifact counts do not include the two specimens assigned to Ua Pou Island at the initial stage of the analysis.

### Island-wide archaeological patterns

**Fig 7:**
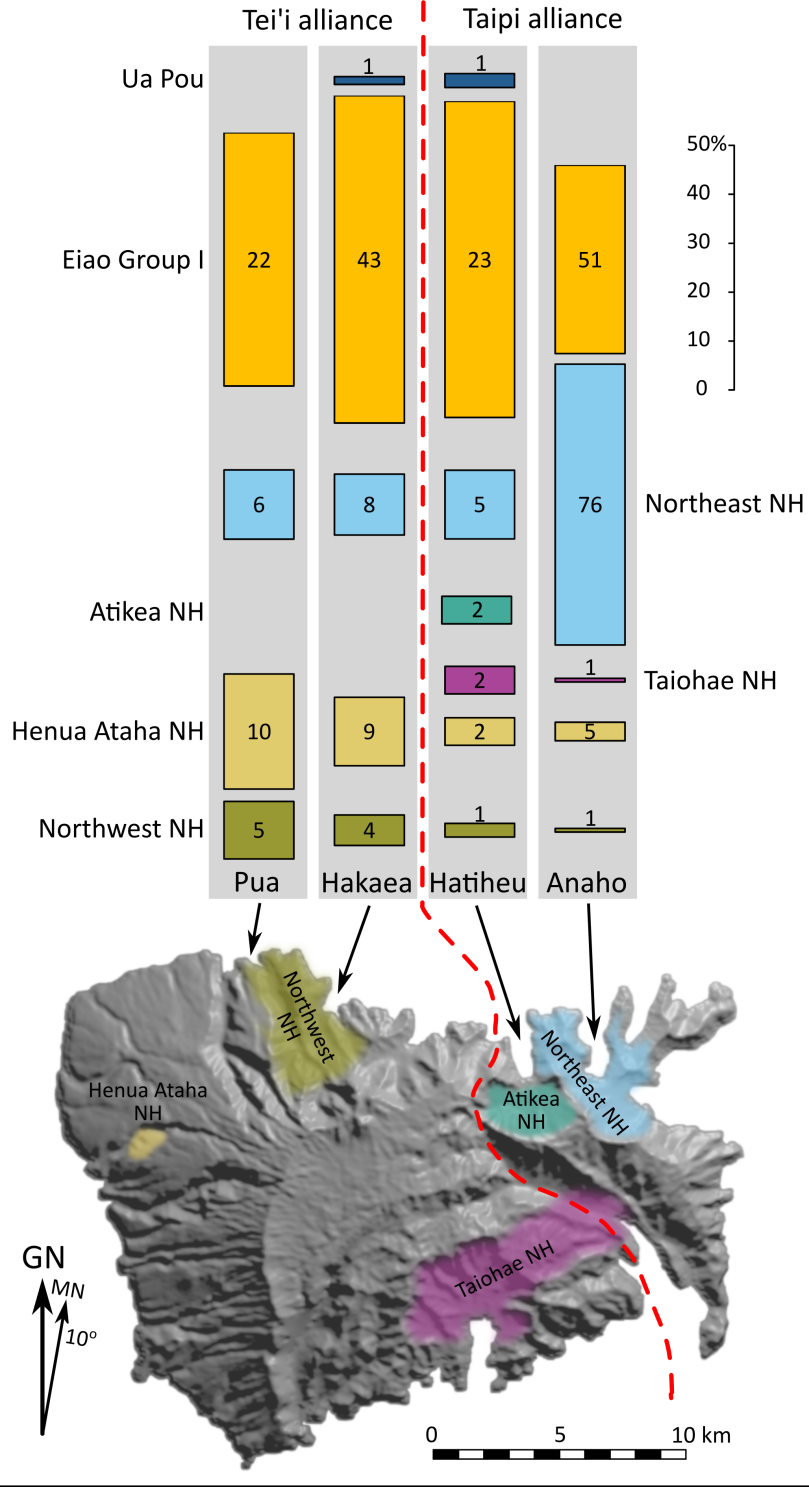
Distributions of adze, chisel, and preform specimens by valley. Bar lengths indicate proportions (%); numbers show counts. The red dashed line shows the approximate supra-tribal alliance boundary as known in the 19^th^ century.

As indicated above, seven geochemical groups, five from Nuku Hiva and two from other Marquesan islands were identified in the archaeological assemblages. Previous work by McAlister [49] did not identify any extra-archipelago sources, consistent with prior Marquesan studies [41,52,53], but differing from many other Polynesian localities, particularly contexts dating to the early regional settlement period [20,21,23,54]. Presumably this is at least partially related to the availability of fine-grained adze-quality stone in abundance on Eiao Island. The five Nuku Hiva geochemical groups include two western sources, two eastern sources, and one southern group (Fig 7, Table 3). Most of these geochemical sources include multiple neighboring quarries and utilized outcrops, which were combined here given their indistinguishable geochemical similarities (see above). Two specimens are geochemically similar to phonolites from Ua Pou Island, c. 30 km to the south. The largest number of off-island tools, however, derive from Eiao Island (50% of the total). Specimens from Northeast Nuku Hiva, a broad geographic area spanning three valleys, constitute 34% of the overall assemblage. The remaining sources each represent less than 10% (Table 3). The results demonstrate that both Nuku Hiva and off-island sources are widely distributed and Eiao tools in particular occur in high frequencies in all four valleys.

**Table 3:** Artifact source assignments by valley. Modes for each source are underlined.

	Valley				
**Source**	**Pua**	**Hakaea**	**Hatiheu**	**Anaho**	**Total**
*Count*					
Eiao Group I	22	43	23	51	139
Northeast NH	6	8	5	76	95
Henua Ataha NH	10	9	2	5	26
Northwest NH	5	4	1	1	11
Taiohae NH			2	1	3
Atikea NH			2		2
Ua Pou		1	1		2
**Total**	**43**	**65**	**36**	**134**	**278**
*Percent*					
Eiao Group I	51.2	66.2	63.9	38.1	50.0
Northeast NH	14.0	12.3	13.9	56.7	34.2
Henua Ataha NH	23.3	13.8	5.6	3.7	9.4
Northwest NH	11.6	6.2	2.8	0.7	4.0
Taiohae NH			5.6	0.7	1.1
Atikea NH			5.6		0.7
Ua Pou		1.5	2.8		0.7

### Functional analysis

Given the reputed quality of Eiao stone, we evaluated the possibility that the widespread distribution of Eiao adzes was necessitated by functional requirements. This was facilitated by our use of whole tools rather than flake debitage. The assemblage was divided into two broad raw material groups, Nuku Hiva versus Eiao, and three morphological traits related to tool function were examined. These included: 1) *cross-section shape*, which correlates with other functional attributes such as bevel width and blade thickness [49,76]; 2) *cross-section area* (cm^2^), taken at the shoulder or mid-section—a useful proxy of adze size as it is less affected by breakage and reworking and can be measured on most specimens (S2 Fig, S2 Table); and 3) *bevel width*, which varies with tool use; narrow bits, for example, concentrate force in a small area, making them ideal for gouging and splitting timbers, while tools with wide bevels are well-suited for dressing and trimming [49,76].

**Fig 8:**
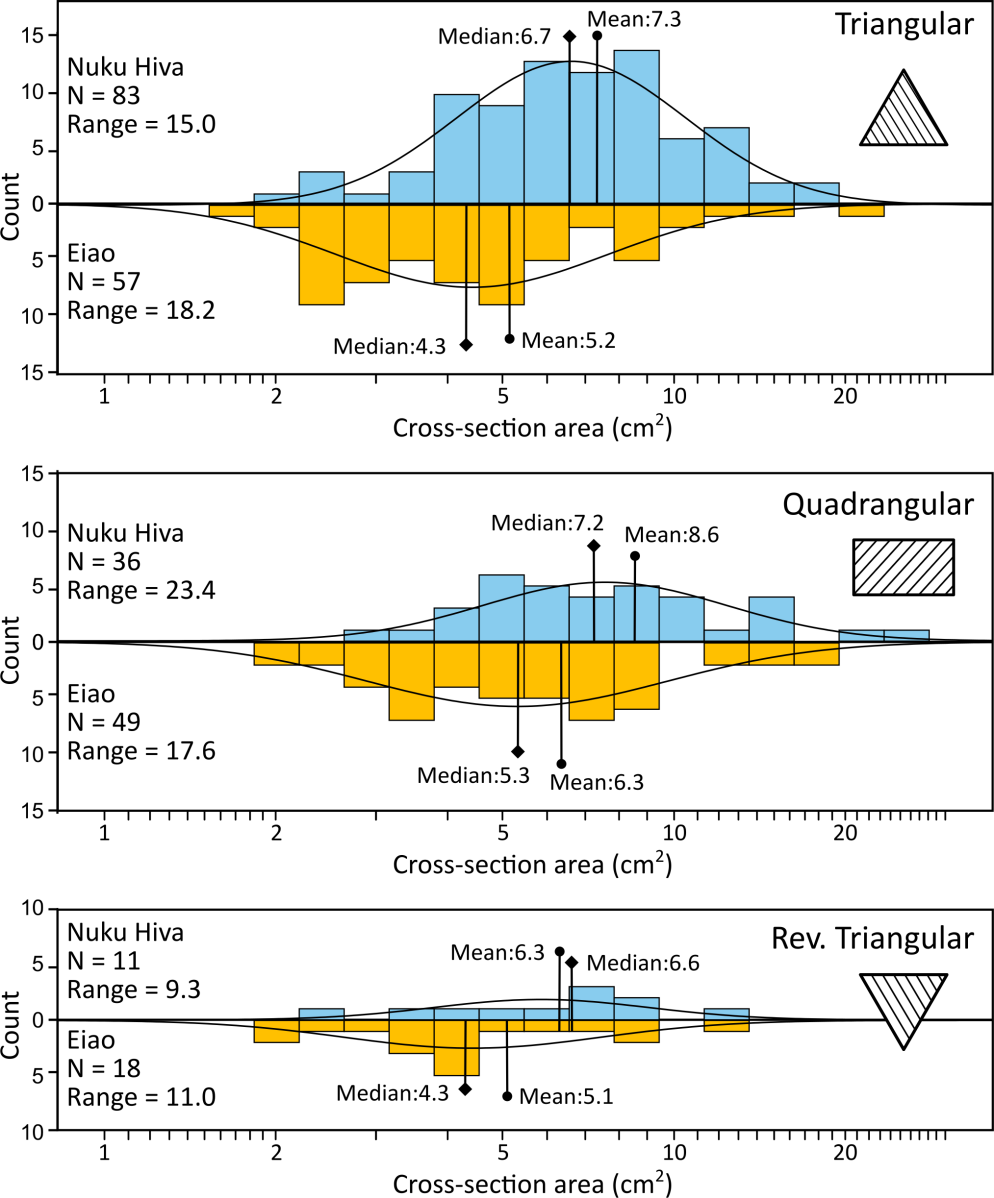
Distributions of cross-section area by source island, separated by cross-section shape. Normal curves are superimposed over the distributions.

Three cross-section shapes, categorized using front-to-back width ratios [49,77], dominate the assemblage: triangular, quadrangular, and reverse-triangular. No definitively early Marquesan adze types were identified, such as the lenticular and plano-convex cross-sectioned Hatiheu and Ha‘e‘eka types described by Suggs and Sinoto [42,78,79], a finding consistent with the late-prehistoric contexts of our assemblages. All cross-section shapes were rendered in both Eiao and Nuku Hiva raw materials (Fig 8, Table 4). Rank order abundances of the different forms are consistent for Eiao and Nuku Hiva stone: triangular forms are the most common, followed by quadrangular, and reverse-triangular. Proportionally, however, triangular adzes are more commonly associated with Nuku Hiva sources (61%), compared to the Eiao source (41%), a difference that is significant at the 0.05 level (χ^2^ = 8.37, df = 2, p = 0.015).

**Table 4:** Adze cross-section shapes by assigned source. Modes for each source are underlined.

	Cross-section shape^a^				
**Assigned Source**	**▲**	**■**	**▼**	**?**	**Total**
*Count*					
**Eiao Group I**	**57**	**49**	**18**	**15**	**139**
**Nuku Hiva Total**	**83**	**36**	**11**	**7**	**137**
Northeast NH	58	24	6	7	95
Henua Ataha NH	18	6	2		26
Northwest NH	5	4	2		11
Taiohae NH	2	1			3
Atikea NH		1	1		2
**Ua Pou**	**1**	**1**			**2**
Total	141	86	29	22	278
*Percent*					
**Eiao Group I**	**41.0**	**35.3**	**12.9**	**10.8**	
**Nuku Hiva Total**	**60.6**	**26.3**	**8.0**	**5.1**	
Northeast NH	61.1	25.3	6.3	7.4	
Henua Ataha NH	69.2	23.1	7.7		
Northwest NH	45.5	36.4	18.2		
Taiohae NH	66.7	33.3			
Atikea NH		50.0	50.0		
**Ua Pou**	**50.0**	**50.0**			
Total	50.7	30.9	10.4	7.9	
*Rank*					
**Eiao Group I**	**1**	**2**	**3**	4	
**Nuku Hiva Total**	**1**	**2**	**3**	4	
Northeast NH	1	2	4	3	
Henua Ataha NH	1	2	3		
Northwest NH	1	2	3		
Taiohae NH	1	2			
Atikea NH		1	1		
**Ua Pou**	**1**	**1**			

^a^ ▲ = Triangular; ■ = Quadrangular, ▼ = Rev. Triangular;? = Indeterminate.

Comparison of cross-section area by raw material type shows that size distributions for Eiao and Nuku Hiva specimens overlap and are approximately log-normal for all cross-section shapes, pointing to a wide and similar range of tool sizes (Fig 8). While there is no significant difference in size for reverse-triangular specimens (t = 1.77, df = 27, p = 0.090), Eiao tools with quadrangular and triangular cross-sections are, on average, smaller than their Nuku Hiva counterparts (p<0.01 for both forms). This could reflect different functional applications, long-distance transport costs, and/or more reworking of a valued raw material.

Ninety-five specimens were complete enough to measure bevel width. Comparison of this attribute by cross-section shape indicates overlapping size ranges for Eiao and Nuku Hiva specimens, with no significant differences in the means (S3 Table). To control for absolute size, an index of bevel width relative to shoulder width was calculated; values less than 1.00 indicate relatively narrow bevels, those greater than1.00 have relatively wide bevels, and those approaching 1.00 constant widths. Again, the ranges overlap and no significant differences were found between Eiao and Nuku Hiva tools (S3 Table), indicating that raw materials from both islands were used to make tools of similar forms, presumably for similar functions.

In sum, while there are differences between tools made from Eiao versus Nuku Hiva stone, the overall pattern is one of similarity. With respect to cross-section shape, all three of the main shapes were rendered in both Eiao and Nuku Hiva raw materials with some frequency. With respect to size, the quadrangular and triangular Eiao adzes are on average smaller than those manufactured from Nuku Hiva stone, but there are no significant differences between absolute or relative bevel widths. These results indicate that Eiao stone was not required for, or restricted to, particular tool forms. While Eiao stone might have been preferred for some tasks, both Eiao and Nuku Hiva sources produced functional tools in the same range of forms.

### Inter-valley patterns

To understand island-wide patterns and the mechanisms associated with non-local material movement, we examined the representation of both Eiao and non-local Nuku Hiva stone across the four valleys. On socio-political grounds Hatiheu Valley was considered the most likely entry point for Eiao tools. Not only is this the largest catchment on this coast, but at Western Contact it was inhabited by several powerful subtribes and it is archaeologically distinguished by multiple large and visually-commanding community and religious complexes [45,80,81].

The evidence, however, indicates that Eiao stone is well represented in all valleys: Pua (51%), Hakaea (66%), Hatiheu (64%), and Anaho (38%). Only Anaho, with three local/nearby stone sources, has a significantly lower proportion of Eiao tools compared to those from Nuku Hiva sources combined (χ^2^ = 18.67, df = 3, p>0.01). Comparison of the other three valleys indicates there are no significant differences in the proportions of Eiao stone (χ^2^ = 3.08, df = 2, p = 0.21). We also examined the proportions of finished to unfinished Eiao tools across all four valleys, with the expectation that direct contact with Eiao would result in a higher percentage of adze preforms or rough-outs. Although Pua has a higher percentage of unfinished tools (59%), the ratio of preforms to finished Eiao adzes across all valleys is not significantly different (χ^2^ = 4.18, df = 3, p = 0.24, S4 Table). In sum, there is no definitive evidence that any given valley acted as an entry point for Eiao Island goods, or subsequently controlled distribution across Nuku Hiva.

The inter-valley distribution of tools from non-Eiao sources, specifically those from Nuku Hiva Island provides further insights. The expectation was that the most proximate sources would be best represented in each valley context. This proposition is supported, with western stone sources predominating in the western valleys and eastern sources in the east (Fig 7). Additionally, the presence of unfinished tools of extra-local raw materials in all valleys shows that at least some Nuku Hiva Island stone was circulating in unfinished forms, similar to the case for Eiao stone.

At the district or supra-valley level (Tei‘i versus Taipi Districts), other patterns emerge. Notably, stone tools made from raw materials derived from Taipi or Tei‘i localities in both cases moved across supra-tribal boundaries. In the western Tei‘i District, Eiao tools represent 60% of the total, compared to 44% for the eastern Taipi District. Additionally, Eiao preforms are better represented in the western valleys (Table 3). Non-Eiao tools from outside the Tei‘i District contribute another 14%, compared to 8% in the Taipi case. Overall, 74% of the Tei‘i District assemblage is derived from non-local stone. Possibly communities in these drier, less fertile valleys had greater incentives to engage in exchange or trade.

Overall, the most important finding from the inter-valley analysis is evidence for a high degree of stone tool movement, with both off-island (Eiao and Ua Pou) and regional (Nuku Hiva) tools and preforms being distributed over considerable distances, between valleys, and across historically recognized socio-political boundaries, even though materials suitable for production of a range of adze forms were available from immediate or nearby sources.

### Intra-valley patterns

To understand intra-valley stone tool distributions we drew on detailed systematic survey data from Anaho Valley, where over 300 architectural structures have been recorded, including primary residential structures (*paepae*) (often with closely associated minor architectural features), agricultural features, storage pits, small shrines, and possible *me‘ae* but, notably, no large community architecture (*tohua*) [43]. Eighty-seven of the structures are consistent with early ethnohistoric and ethnographic descriptions of primary residential house foundations (*paepae*) that were the focus of many household-level domestic activities and were used for sleeping [43,59]. Specifically these are dry stone masonry terraces and platforms larger than 20 m^2^, often internally divided, and sometimes with stone-lined pits, postmolds, and small adjoining pavements. Excavation, dating, and comparison with examples from elsewhere indicate that these residential structures are largely if not solely late prehistoric to early historic in age, specifically post-dating AD 1650 [43]. The Anaho primary residential structures fall into two size classes: numerous small foundations (mainly between 30 and 60 m^2^) and a limited number of medium-to-large foundations (90 to 174 m^2^).

The medium-to-large examples of primary residential features are distinguished from smaller examples not only by their size, but also by mega-boulder (>1.5 m in across) facing stones (common), imported red tuff facing slabs (occasional), and distinctive artifacts (rare, but excavations have been limited). The larger examples are typically associated with smaller auxiliary features, such as large food storage pits and smaller structures that differ from house foundations in terms of size and form [42,43,59]; some of the latter are likely to be shrines and cooking areas. As Feinman and Neitzel suggest [82], the most compelling indication that the large terraces and platforms were elite residences lies in the labor required for their construction; both the mega-boulder facings and the sheer volume of rock required for fill, suggest occupants of rank who commanded significant authority over labor resources within the local community (see also [42]). Earle [83] further argues that residential sites are a particularly useful measure of the differentiation of wealth and social inequality, as “housing involves a daily use and display function … likely to represent economic and political relationships …” (pg. 219).

The primary residential structures of Anaho are widely distributed across the valley but form seven clusters, each covering several hundred square meters, and presumably representing several related households [43]. Notably in each cluster there is only one large and sometimes one medium structure. The dispersed distribution of the medium-to-large structures across the valley, their ornamentation, and their associations with communal-size storage pits are consistent with criteria used to identify elite sites elsewhere in Polynesia [24,84–86].

Seventy-seven of the artifacts derive from on or in the vicinity of 19 of these late prehistoric domestic structures (Table 5). The expectation was that imported Eiao tools would be preferentially associated with the medium-large residential complexes, as in other Polynesian case studies. Importantly, Eiao stone is sufficiently distinctive that it would not have been mistaken with materials from other sources by indigenous Marquesans [47]. However, when the proportions of Eiao adzes are compared to those from the Nuku Hiva sources combined, the differences across small versus medium-to-large structures are not significant (p = 0.35, Fisher's exact test, one-tailed). Smaller samples of stone tools from domestic complexes in the other three valleys are not amenable to statistical analysis but show similar patterning. Overall, the relationship between structure size and proportions of Eiao stone tools is not significant (an unanticipated finding in light of acquisition costs); this result is further considered below.

**Table 5:** Frequencies of tools associated with small and medium/large domestic structures by assigned source.

	Structure size	
**Assigned** **Source**	**Small** **(<60m2) N = 12**	**Medium/Large** **(>60m2) N = 7**
*Count*		
**Eiao Group I**	**19**	**15**
**Nuku Hiva Total**	**21**	**22**
Northeast NH	19	19
Henua Ataha NH	2	2
Northwest NH		1
**Total**	**40**	**37**
*Percent*		
**Eiao Group I**	**47.5**	**40.5**
**Nuku Hiva Total**	**52.5**	**59.5**
Northeast NH	47.5	51.4
Henua Ataha NH	5.0	5.4
Northwest NH		2.7

## Discussion

This analysis is the largest and most comprehensive Marquesan stone tool geochemical study to date, and provides a foundation for comparisons with results from other chiefdoms and archaic states where exchange had an important socio-political role. The prominence of exchange in late Marquesan prehistory is clearly evident from our results: adzes from at least seven distinct Marquesan sources were widely distributed, with an off-island source (Eiao Island) being especially well represented (around 50%). Importantly, a functional analysis shows that Eiao stone was not required for specific tool forms, indicating that social (not utilitarian) processes probably played a major role in structuring tool distributions. Data from four valleys and two supra-tribal districts further demonstrate that both preforms and finished tools of non-local (extra-valley) stone were widely distributed, and the frequency of exchange was generally similar across the four catchments. An intra-valley analysis, using the well-studied catchment of Anaho, sought to determine if non-local tools were preferentially associated with elite structures (defined by size, ornamentation, auxiliary features, and other criteria) but, contrary to expectations, this is not the case. Overall, the dominant patterns to emerge from our analysis are high abundances and broad dissemination of non-local tools at multiple scales: across a traditionally recognized supra-tribal boundary, between valleys, and across social classes within Anaho Valley. Our findings unambiguously point to the importance of social networks in these low hierarchy polities, but determining how stone resources moved within and between areas is not straight-forward.

As discussed earlier, recent studies have shown that non-local stone tools are strongly associated with elite activities in some of Polynesia’s most highly stratified polities (e.g., those of Hawai‘i, Tonga, and the Society Islands) [7,8,24]. In these examples, material wealth was accumulated by powerful, established elite hierarchies through tribute, taxation, and direct control over scarce and unevenly distributed resources. In the Marquesas, in contrast, formal taxation was lacking and political integration weak, generally being confined to one or two adjacent valleys—at least at Western Contact. Early ethnohistoric observations suggest that different mechanisms and diverse cultural motivations promoted exchange of a variety of specialty goods in the Marquesas [33,35,87]. These goods fall into three broad categories: a) scarce natural resources (e.g., feathers, canoe timbers, sperm whale teeth); b) manufactured goods (e.g., stone food pounders, porpoise tooth headdresses, various carved ornaments, prepared turmeric, and adzes); and c) agricultural produce, especially pigs. Further, individual valleys as well as entire islands were associated with particular niche products [35,36,38,87,88], suggesting endeavors to establish desirable products that might be monopolized. At Contact such items were exchanged in a variety of contexts, including competitive inter-tribal feasts, community festivals, betrothals, resolution of disputed lands, and formal name exchanges (i.e., *o inoa*, a social institution that granted individuals shared rights and privileges [38]. Exchange goods thus served as political currency for elites, who deployed them in complex webs of rivalry and cooperation to garner labor and support, and create alliance networks [89]. In these respects, Contact-period Marquesan polities had much in common with emergent hierarchies and prestige chiefdoms elsewhere [83,89–92].

The archaeological record of Marquesan stone tools assembled here is in many ways consistent with the exchange patterns suggested by ethnohistoric observations. However, archaeologically determining whether adze production and distribution took place under formal elite control, or via informal sharing between individuals and households, can be challenging. Winterhoff [11], for instance, has argued for increasing control of adze production by Tutuila chiefs during late Sāmoan prehistory (ca. post-1000 AD), largely on the basis of strategic positioning of adze production centers. However, low frequencies of artifact transfer within Sāmoa hinder detailed distributional analyses [11,93]. At the large, high-altitude Mauna Kea Adze Quarry of Hawai‘i inaccessibility, provisioning requirements, and evidence of large-scale logistical and spatial organization within the quarry complex have been interpreted as evidence of elite control over tool production, although island-wide tool distribution patterns have led some to argue otherwise [94–98]. On Rurutu (Austral Islands), elite associations with adze production and distribution are incontrovertible, with the important Vitaria Quarry and adze production zone lying in the heart of a highly nucleated chiefly center where it is surrounded by elite residences, a major chiefly meeting ground, and the important ritual site of Marae Tararoa [10]. Indeed, Rolett and colleagues argue that “the value of Vitaria stone and adzes may have contributed to the rise of Vitaria’s extraordinary urban complex and chiefly center” (pg.470).

The case for elite control of the Eiao Island stone resources is less straightforward but nonetheless suggestive. Foremost, inter-island transport costs would have been high. Seaworthy vessels were required for the 100-km open-ocean voyage between Nuku Hiva and Eiao, a sometimes treacherous crossing, with at least one chief being lost at sea while en route to the island [32]. Canoes suitable for blue water sailing are costly to build and elsewhere in Polynesia voyaging canoes were typically controlled by high-ranking individuals. Early Contact-period accounts of Marquesan society report that elites and propertied individuals owned not only voyaging canoes, but also simple fishing vessels [32,99]. Elite involvement on Eiao Island also is indicated by large ritual structures (*me‘ae*) traditionally associated with powerful chiefs and priests, and of a size that would have required significant organized labor [44,50]. Linton’s early 20^th^ century informants recalled that Eiao was under the control of one or more Nuku Hiva tribes, while Handy was told that Eiao was once a place of chiefly burial [38]. Conceivably, Eiao’s stone quarries could have been an open access resource early in the Marquesan settlement period, when Eiao tools were widely distributed across East Polynesia. However, this seems unlikely in late prehistory given Contact-period accounts of elite controls and resource ownership, cultural regulations on commoners, and Marquesan socio-political organization in general [36].

The mechanisms by which Eiao and other non-local Nuku Hiva stone tools circulated between valleys also are of interest. Archaeological studies suggest that adzes typically left Eiao Island as unfinished rough-outs [47,100] and consistent with these accounts, both preforms and finished adzes were recovered from the four Nuku Hiva valleys sampled here. The relatively similar frequencies of Eiao tools across these four catchments suggest there was no centralized control at the island or supra-tribal scale, and elites in each valley could have operated independently in the acquisition and subsequent dissemination of Eiao materials. However, Nuku Hiva Island stone tools, although less abundant, provide an interesting contrast. Despite the widespread availability of suitable fine-grained basalts, stone from all of the major Nuku Hiva sources documented in our study occurs in most valleys and frequencies approximate expectations for localized control with down-the-line exchange [101].

Most challenging are questions of how and why imported tools came to be so well represented in not only elite but also commoner residential sites in our single valley-scale analysis. Assuming elites provisioned Eiao expeditions, as argued above, it is likely that they also controlled subsequent distribution of this costly and valued resource. Mills and Lundblad [97] suggest that patterns of access and distribution can be illuminated by combining geochemical analyses with technological studies of tool reduction. Although technological studies are not available for Anaho, two of the largest residential complexes in the valley have evidence that is suggestive of preform reduction and adze finishing, specifically abundant flaking debris and, in one case, grinding stones. While direct access to non-local (extra-valley) stone sources by Anaho’s commoners is theoretically possible, this is inconsistent with what is known about Marquesan society and socio-political structures as observed at Western Contact; additionally this model requires several assumptions about fundamental socio-political change between late prehistory and the early Contact-period. An alternative possibility is that adzes manufactured from costly imported stone made their way into commoner households as tools approached the end of their use-lives; however, this is not supported by the evidence at hand. In our view the most parsimonious explanation is that the stone tool patterns observed at Anaho are an empirical manifestation of 18^th^ century ethnohistorical accounts of exchange and socio-political relations within and between Marquesan social classes [32,33,35,36]. Those accounts highlight how elites strategically used a variety of wealth goods and staple resources to differentiate themselves from political competitors and attract followers in a society where chiefly sanctity and genealogical links were diminished, and socio-political power and authority were contested and dynamic. We thus suggest that Nuku Hiva elites controlled the importation of non-local stone tools and distributed them to commoners and craft specialists in return for political support, labor, and agricultural produce, and for use in the production of secondary goods (e.g., canoes, carvings, and ornaments)—all which could be directed to prestige rivalry, alliance building, and elite competition in varied arenas. Ultimately other evidence is needed to fully explain the mechanisms responsible for the patterns of imported tools across elite and commoner sites as observed in Anaho Valley. This might include not only technological studies, but also samples from ceremonial and ritual contexts, and data from other valleys to determine whether the Anaho situation is representative of broader patterns.

Returning to regional comparisons, the high proportion of non-local (extra-valley and off-island) tools identified by our analysis, averaging 77% across the four valleys, approximates or exceeds findings from more strongly hierarchical societies elsewhere. In the archaic Hawaiian state of Kahikinui District, adzes and flakes from non-local (external to the district) sources comprised 27% of the combined residential and ritual assemblages (and 44% of the latter) [24]. Even higher frequencies were observed in elite activity areas of the Tongan maritime system (66% extra-archipelago) [7]. In contrast, our Marquesan study found that non-local materials occurred in nearly equal proportions on elite (49%) and commoner (53%) residential sites. These results also contrast with the Kahikinui residential data (24% non-local) [24], with assemblages from domestic contexts on Mo‘orea, Society Islands (33% non-local) [8], and with results from pre-state Tongan contexts where 33% of the adzes-flakes were non-local [7]. Overall, although specific aspects of our findings are unexpected, they nonetheless confirm the importance of exchange in Polynesian chiefdoms of variable political complexity.

## Conclusions

The Marquesan case study offers insights into the roles played by wealth goods and exchange in the evolution of socio-political complexity. Of particular interest here are contrasts between patterns of exchange in low hierarchy, weakly centralised tribal societies and those of strongly hierarchical chiefdoms and archaic states. Our analysis reveals a high frequency and widespread distribution of non-local stone tools across a broad geographic region in late prehistory: the northern coast of Nuku Hiva Island. Importantly, we demonstrate that exchange was not necessitated by the unavailability of suitable local raw materials or by tool functional requirements, but rather was more likely driven by social processes. The identified tool raw materials derive from both off-island (Eiao and Ua Pou) sources and extra-valley provenances in frequencies that approach, and sometimes exceed, those observed in more centralized and hierarchical Polynesian societies. Our analysis suggests that the character and role of exchange co-evolved with other processes that drove increasing social inequality, socio-political hierarchy, and centralization, but the relationships were complex and not necessarily linear. In the Marquesan case, the importance of exchange is clearly indicated, but the specific mechanisms by which non-local stone tools moved within and across valleys are not fully resolved.

While our study highlights that Polynesian exchange was variable and probably driven by diverse causal processes, comparisons with other localities have not been straight-forward. Differences in the kinds of materials analyzed (whole tools versus flakes), variation in the functional contexts that were sampled (residential, ritual, burial, and agricultural) and ways in which “imported” is defined (off-island versus non-local to the catchment or socio-political unit under study) complicate direct cross-site comparisons. Nonetheless, our results demonstrate the potential of stone tool geochemistry to inform on variation in Polynesian exchange systems and the role of exchange and interaction in socio-political processes, within the region and beyond.

## Supporting information

S1 Appendix XRF calibration details. (PDF)

S1 Dataset Calibrated WDXRF and EDXRF data. (XLSX)

S1 Fig Scatterplots of the node divisions for the classification tree analysis. Reference specimens are shown on the right and artifacts on the left. Support vector machine decision boundaries are indicated with solid lines and support vector margins with dashed lines.
(TIFF)

S2 Fig Scatterplot showing the relationship between weight and cross-section area for complete adzes and preforms. The regression line is for all cross-section shapes combined. Data are from [49].
(TIFF)

S1 Table Summary data for discriminant function analyses. (DOCX)

S2 Table Summary statistics for correlations between weight (g) and shoulder cross-section area (cm^2^) for complete adzes and preforms. (DOCX)

S3 Table Comparison of absolute and relative bevel width by cross-section shape and source island. (DOCX)

S4 Table Frequencies of artifacts sourced to Eiao Island by manufacturing stage. Finished adzes were identified by the presence of polish on one or more surface.
(DOCX)
