# Supplementary material for: Basalt geochemistry reveals high frequency of prehistoric tool exchange in low hierarchy Marquesas Islands (Polynesia)
Source: PLoS One. 2017 Dec 27;12(12):e0188207. doi: 10.1371/journal.pone.0188207 (PMC5744946; doi:10.1371/journal.pone.0188207)
Supplement: S1 Appendix — (PDF) [file pone.0188207.s001.pdf]

S1 Appendix: XRF calibration details

Two analytical methods were used in this study—Wavelength Dispersive X-ray Fluorescence (WDXRF) and Energy Dispersive X-ray Fluorescence (EDXRF). The former is more accurate and measures a greater range of elements but it is also partially destructive, requiring at least 2g of powdered rock, so is not suitable for artefacts that are very small or too valuable to be physically altered. For this reason non-destructive EDXRF also was employed. All archaeological and reference specimens (n=541) were analyzed using EDXRF. In addition, a portion of the sample (n=67) was subjected to WDXRF analysis as a check on the validity of the EDXRF calibration and to provide a sample of fully-compositional data. We note that the majority of the specimens included in this study were previously analyzed by McAlister [1] using an older generation Alpha 4000 portable EDXRF analyzer equipped with a solid-state silicon photodiode (Si-PIN) detector. These specimens have been reanalyzed here with a more advanced Bruker Tracer III SD EDXRF analyzer equipped with a silicon drift detector (SDD).

Wavelength Dispersive X-ray Fluorescence (WDXRF). The WDXRF analysis was conducted at the University of Auckland, School of the Environment Geology Laboratory. A small plug was cut from each selected specimen using a 6mm diameter diamond coring drill bit. The plugs were cut into two pieces and one portion of each sample was ground flat and retained for EDXRF analysis (see below). The other portion was prepared for WDXRF following the standard procedures used by the Geology Laboratory [2,3]. Samples were ground to a homogenous powder of 60 mesh using a ring grinder fitted with a tungsten carbide head. They were then dried at 105°C overnight, followed by ignition at 950°C for two hours. Weights were recorded before and after these procedures to determine loss of water (H_2O-) and loss-on-ignition (LOI). Following ignition, the specimens were weighed to 2.0000g and mixed with 4.0000g of Norrish 12/22 flux. This mixture was then fused into discs using a Fusion Technologies Phoenix 4000 automated casting machine. WDXRF determinations of the fusion discs were carried out by technician, John Wilmshurst, using a Siemens SRS 3000 sequential X-ray spectrometer fitted with an Rh tube. The WDXRF analysis quantified 27 elements: ten major elements as oxides (wt. %), and 17 trace elements in parts-per-million (ppm) (Table A). The Geology Laboratory calibrates its equipment regularly using international rock standards, BR, MRG, JB-1, W-1, AGV-1, JG-1, GH, GA, G-2, SY-2, NIM-S, MIN-L and BCS-375, and a set of internal standards [2], as well as participating in inter-laboratory quality assurance testing [4-6]. The WDXRF results are reported in S1 Dataset.

Energy Dispersive X-ray Fluorescence (EDXRF). The non-destructive EDXRF analyses were carried out using a Bruker Tracer III SD portable analyzer. The instrument employs an X-ray tube with an Rh target and a $10mm^2$ silicon drift detector (SDD), with a typical resolution of 145eV at 100,000cps. The X-ray tube was operated with a setting of 40keV at 10.7μA, in an air-path and through a window composed of 12mil Al and 1mil Ti filters (Bruker's Yellow filter). The Tracer III SD is supplied with in-built factory calibrations for obsidians and fine-grained rock (Bruker's "Obsidian" and "Mudstone" calibrations). However, the manufacturer recommends creating custom calibrations to optimize results for specific materials. A set of 33 references was used for the calibration, including 16 international standards (AGV-2, BIR-1a, BCR-2, DNC-1, GA, GSP-2, JA-1, JA-2, JB-1b, JB-2, JB-3, JG-1a, MRG-1, NIST-688, QLO-1, SY-2) in pressed powder pellet form and 17 oceanic island basalt specimens from the Marquesas Islands, Cook Islands and New Zealand analyzed in cut whole rock form. The oceanic basalt specimens were analyzed at the University of Auckland, School of the Environment Geology Laboratory using Wavelength Dispersive X-ray Fluorescence (WDXRF). They were included both to ensure that similar compositional ranges and elemental ratios as the artefacts were contained within the calibration procedure and also to check whether any systematic error was introduced by analyzing whole rock samples using calibrations based on pressed powder standards.

Concentrations were calculated as oxide weight percentages (wt%) for major elements and as parts-per-million (ppm) for trace elements (including Mn) using Bruker's S1CalProcess (ver. 2.2.33) software. Calibration standards, source reference samples and artefacts were analyzed three times, each for 120 seconds and the results averaged. Selected standards were run each time the instrument was operated to check for drift. Eighteen elements were included in the EDXRF calibration (SiO_2, K_2O, CaO, TiO_2, V, Cr, Mn, Fe_2O_3, Ni, Cu, Zn, Pb, Th, Rb, Sr, Y, Zr, Nb). The calibration results are shown in Fig A. Overall, accurate results were obtained for most elements. In particular, for the mid-Z trace elements (Rb to Nb) most international standards calibrated to within the manufacturers' reported uncertainties. No systematic differences were observed between the pressed powder and whole rock samples.

The EDXRF analyses were carried out on flaked or polished surfaces of both the reference samples and artifacts. Specimens were analyzed three times, each on different portions of their surfaces, and the results were averaged. Because our assemblage consists of relatively large adzes, chisels, and preforms (as opposed to flakes), and the EDXRF beam area is small ($10mm^2$), there was generally some choice in selecting reasonably flat polished or flaked surface areas to analyze.

Studies have shown that, in non-destructive EDXRF analyses, the lighter elements are more susceptible to analytic error caused by weathering and irregular surface geometry [7-9]. Our own research found that some of the lighter elements, particularly SiO_2, V and Cr, exhibited considerable variability across the

surface of the same sample, likely in part because of irregular surface geometry; these elements, and also Pb and Th, which occur only in low concentrations, were not used in the analyses.

As noted above, specimens were analyzed in an air-path using a filter that attenuates the lighter elements' characteristic energy peaks to increase sensitivity for the mid-Z elements. The Tracer III SD is equipped with an optional vacuum chamber, and employing this on unfiltered spectra improves the count rate of the aforementioned light elements. A calibration using the vacuum chamber with no filter was trialed. It halved the errors for SiO_2 (from 2.3% to 1.0% RMSE) and enabled Al_2O_3 to be quantified with an error of approximately 0.5% RMSE but made little difference to the calibrations of the other lighter elements (K_2O – Fe_2O_3T). In the present case where all specimens are Oceanic Island Basalts with similar concentrations of SiO_2 and Al_2O_3, the additional time required for resetting the instrument's parameters and re-analyzing each sample, was not justified. The EDXRF results for the reference samples and artefacts are provided in S1 Dataset.

Comparison of methods. To check for differences between the two methods, the means and standard deviations of the WDXRF and EDXRF analyses for the four most numerous geochemical groups (Eiao Group I, Northeast NH, Henua Ataha NH, and Northwest NH) were compared (Table B). The EDXRF data exhibit more within-group variability, particularly for the lighter elements between SiO_2 and Fe_2O_3T, reflecting the lower precision of that technology. The group means for the two methods, however, are in close agreement.

The validity of portable EDXRF analyzers has been debated in archaeological literature [10,11], but as demonstrated here and elsewhere [12], when appropriately calibrated and used according to manufacturers' recommendations, instruments using current technology are capable of providing accurate analytical data, albeit for a limited range of elements.

Table A. Detection, determination and quantitation limits for WDXRF analysis on fusion discs. Data supplied by John Wilmshurst, University of Auckland, School of the Environment Geology Laboratory.

Element	LLD(1) *(ppm)*	LoD(2) *(ppm)*	LoQ(3) *(ppm)*	Element	LLD(1) *(ppm)*	LoD(2) *(ppm)*	LoQ(3) *(ppm)*
SiO_2	89	177	295	Sc	2.8	6	9
TiO_2	12.8	26	43	V	3.1	6	10
Al_2O_3	58	116	193	Cr	3.5	7	12
Fe_2O_3	6.9	14	23	Ni	2.5	5	8
MnO	4.1	8	14	Cu	2.8	6	9
MgO	31	61	102	Zn	1.9	4	6
CaO	21	42	70	Ga	1.9	4	6
Na_2O	36	73	121	Rb	1.3	3	4
K_2O	7.1	14	24	Sr	1.0	2	3
P_2O_5	8.0	16	27	Y	1.0	2	3
				Zr	1.0	2	3
				Nb	1.0	2	3
				Ba	8.2	16	27
				La	4.8	10	16
				Ce	12.9	26	43
				Pb	3.1	6	10
				Th	1.8	4	6

1) Lower limit of detection
2) Limit of determination
3) Limit of quantitation

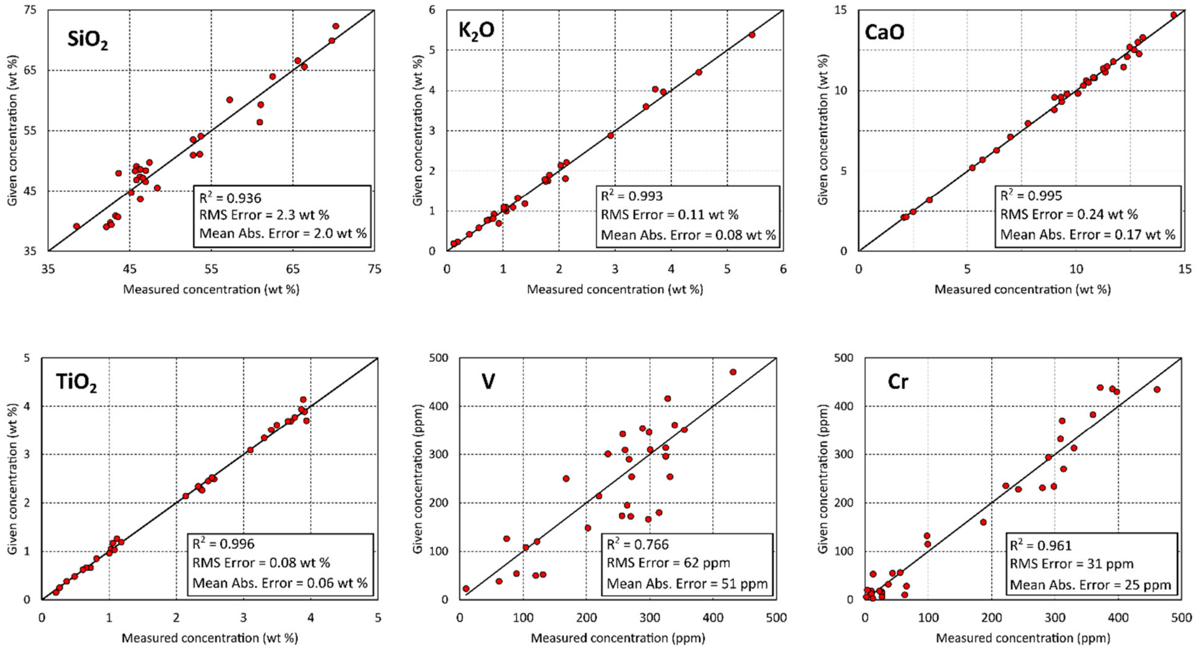

Fig A. Results for the Bruker Tracer III SD calibration. Values for the coefficient of determination (R^2), the Root Mean Square (RMS) Error, and Mean Absolute Error are shown.

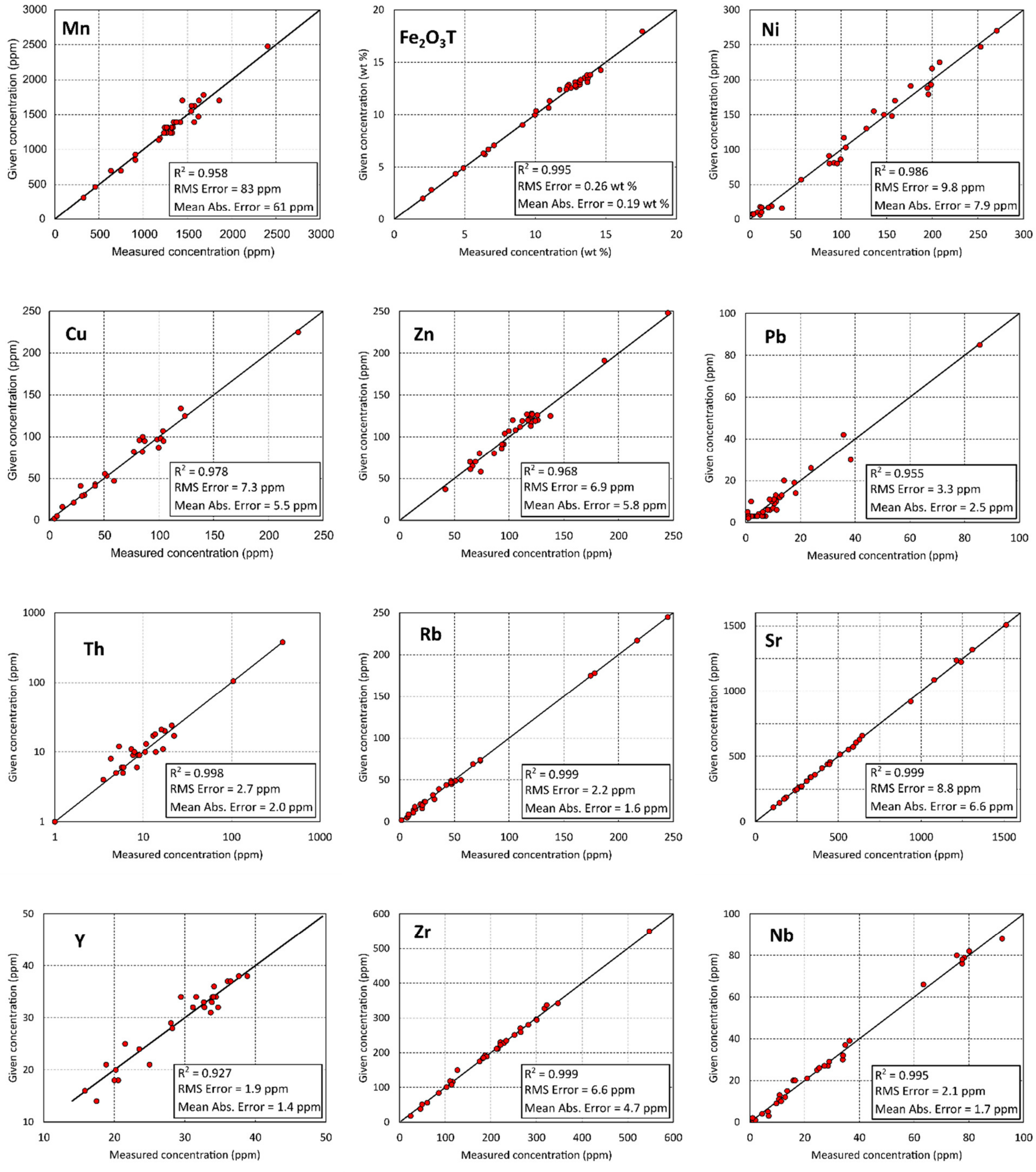

Fig A (continued).

Table B. Summary of WDXRF and EDXRF concentrations for reference samples and archaeological samples assigned to the four main geochemical groups.

	Geochemical Group															
	Eiao, Group I				Northeast Nuku Hiva				Henua Ataha				Northwest Nuku Hiva			
	WDXRF (*n=23*)		EDXRF (*n=116*)		WDXRF (*n=28*)		EDXRF (*n=225*)		WDXRF (*n=8*)		EDXRF (*n=30*)		WDXRF (*n=6*)		EDXRF (*n=36*)	
	**Mean**	*S.D.*	**Mean**	*S.D.*	**Mean**	*S.D.*	**Mean**	*S.D.*	**Mean**	*S.D.*	**Mean**	*S.D.*	**Mean**	*S.D.*	**Mean**	*S.D.*
**SiO_2 (%)***	**46.64**	*0.24*	**46.81**	*1.47*	**48.53**	*0.60*	**47.62**	*2.14*	**46.71**	*0.24*	**47.92**	*1.50*	**47.42**	*0.51*	**46.99**	*1.70*
**K_2O (%)***	**1.02**	*0.04*	**1.11**	*0.10*	**0.86**	*0.16*	**0.78**	*0.20*	**1.13**	*0.07*	**1.06**	*0.13*	**1.14**	*0.20*	**1.03**	*0.27*
**CaO (%)**	**9.26**	*0.05*	**9.32**	*0.21*	**11.05**	*0.37*	**10.87**	*0.83*	**9.96**	*0.11*	**9.92**	*0.21*	**10.63**	*0.30*	**10.80**	*0.43*
**TiO_2 (%)**	**3.87**	*0.02*	**4.12**	*0.22*	**3.49**	*0.22*	**3.68**	*0.36*	**3.43**	*0.02*	**3.40**	*0.15*	**3.97**	*0.20*	**4.13**	*0.27*
**V (ppm)***	**296**	*4*	**298**	*52*	**331**	*22*	**312**	*45*	**321**	*5*	**331**	*36*	**338**	*9*	**338**	*38*
**Cr (ppm)***	**67**	*12*	**95**	*91*	**165**	*93*	**190**	*108*	**340**	*22*	**365**	*66*	**87**	*36*	**107**	*87*
**MnO (%)**	**0.167**	*0.004*	**0.153**	*0.013*	**0.169**	*0.010*	**0.165**	*0.016*	**0.170**	*0.005*	**0.170**	*0.011*	**0.162**	*0.004*	**0.162**	*0.012*
**Fe_2O_3T (%)**	**13.46**	*0.15*	**13.05**	*0.48*	**12.89**	*0.38*	**12.63**	*0.66*	**13.09**	*0.19*	**13.10**	*0.41*	**13.18**	*0.36*	**13.19**	*0.64*
**Ni (ppm)**	**92**	*9*	**97**	*20*	**93**	*22*	**99**	*22*	**221**	*16*	**211**	*17*	**87**	*13*	**102**	*21*
**Cu (ppm)**	**46**	*6*	**44**	*9*	**103**	*11*	**82**	*25*	**66**	*6*	**68**	*12*	**95**	*6*	**96**	*11*
**Zn (ppm)**	**126**	*4*	**126**	*9*	**112**	*4*	**113**	*8*	**114**	*4*	**116**	*5*	**122**	*6*	**123**	*9*
**Pb (ppm)***	**3**	*2*	**6**	*2*	**2**	*2*	**7**	*3*	**3**	*1*	**6**	*2*	**3**	*1*	**5**	*3*
**Th (ppm)***	**10**	*2*	**9**	*2*	**9**	*3*	**7**	*2*	**10**	*1*	**8**	*1*	**12**	*2*	**9**	*2*
**Rb (ppm)**	**21**	*3*	**25**	*3*	**20**	*5*	**20**	*6*	**27**	*2*	**30**	*4*	**24**	*6*	**24**	*7*
**Sr (ppm)**	**599**	*5*	**606**	*8*	**446**	*47*	**446**	*48*	**482**	*8*	**483**	*12*	**574**	*34*	**569**	*42*
**Y (ppm)**	**37**	*1*	**36**	*2*	**33**	*3*	**33**	*4*	**35**	*4*	**33**	*4*	**37**	*2*	**36**	*2*
**Zr (ppm)**	**293**	*5*	**295**	*7*	**250**	*23*	**240**	*23*	**249**	*9*	**251**	*12*	**316**	*22*	**309**	*17*
**Nb (ppm)**	**27**	*1*	**28**	*2*	**25**	*4*	**25**	*5*	**30**	*1*	**31**	*4*	**35**	*2*	**33**	*4*

* element not used in the analyses.

References

1. McAlister A (2011) Methodological issues in the geochemical characterisation and morphological analysis of stone tools: A case study from Nuku Hiva, Marquesas Islands, East Polynesia [PhD thesis]. Auckland: University of Auckland.
2. Parker RJ, Sheppard PJ (1997) Pacific island adze geochemistry studies at the University of Auckland. In: Weisler MI, editor. Prehistoric long-distance interaction in Oceania: an interdisciplinary approach. Auckland: New Zealand Archaeological Association, Monograph 21. pp. 205–211.
3. Parker RJ, Smith IEM, Wilmshurst JK (1993) Sample Preparation for XRF and ICP analysis. Department of Geology, University of Auckland, Report No 2.
4. Thompson M, Potts PJ, Kane JS, Chappell BW (1999) GeoPT3. International proficiency test for analytical geochemistry laboratories - Report on Round 3. Geostandards Newsletter 23: 87–101.
5. Thompson M, Potts PJ, Kane JS, Webb PW, Watson JS (1998) GeoPT2. International proficiency test for analytical geochemistry laboratories - Report on Round 2. Geostandards Newsletter 22: 127–156.
6. Thompson M, Potts PJ, Kane JS, Webb PW (1996) GeoPT1. International proficiency test for analytical geochemistry laboratories - Report on Round 1. Geostandards Newsletter 20: 295–325.
7. Gauthier G, Burke AL (2011) The effects of surface weathering on the geochemical analysis of archaeological lithic samples using non-destructive polarized energy dispersive XRF. Geoarchaeology 26: 269–291.
8. Lundblad SP, Mills PR, Hon K (2008) Analysing archaeological basalt using non-destructive energy-dispersive x-ray fluorescence (EDXRF): effects of post-depositional chemical weathering and sample size on analytical precision. Archaeometry 50: 1–11.
9. Ogburn D, Sillar B, Sierra JC (2013) Evaluating effects of chemical weathering and surface contamination on the in situ provenance analysis of building stones in the Cuzco region of Peru with portable XRF. Journal of Archaeological Science 40: 1823–1837.
10. Frahm E (2014) Characterizing obsidian sources with portable XRF: accuracy, reproducibility, and field relationships in a case study from Armenia. Journal of Archaeological Science 49: 105–125.
11. Shackley MS (2010) Is there reliability and validity in Portable X- ray Fluorescence Spectrometry (PXRF)? SAA Archaeological Record 10: 17–20.
12. Conrey R, Goodman-Elgar M, Bettencourt N, Seyfarth A, Van Hoose A, Wolff J (2014) Calibration of a portable X-ray fluorescence spectrometer in the analysis of archaeological samples using influence coefficients. Geochemistry: Exploration, Environment, Analysis 14: 291-301.
