# Supplementary figures and images for: Basalt geochemistry reveals high frequency of prehistoric tool exchange in low hierarchy Marquesas Islands (Polynesia)
Source: PLoS One. 2017 Dec 27;12(12):e0188207. doi: 10.1371/journal.pone.0188207 (PMC5744946; doi:10.1371/journal.pone.0188207)

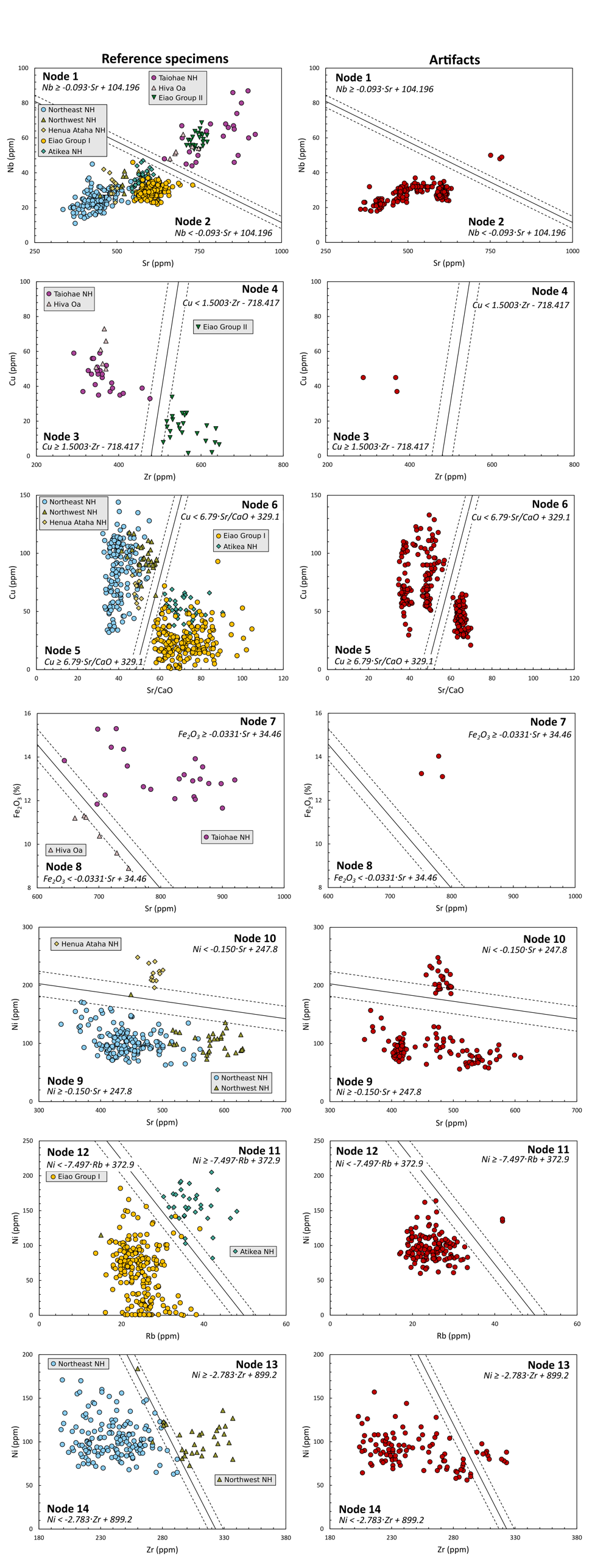

Supplement: S1 Fig — Reference specimens are shown on the right and artifacts on the left. Support vector machine decision boundaries are indicated with solid lines and support vector margins with dashed lines. (TIFF) [file pone.0188207.s003.tiff]

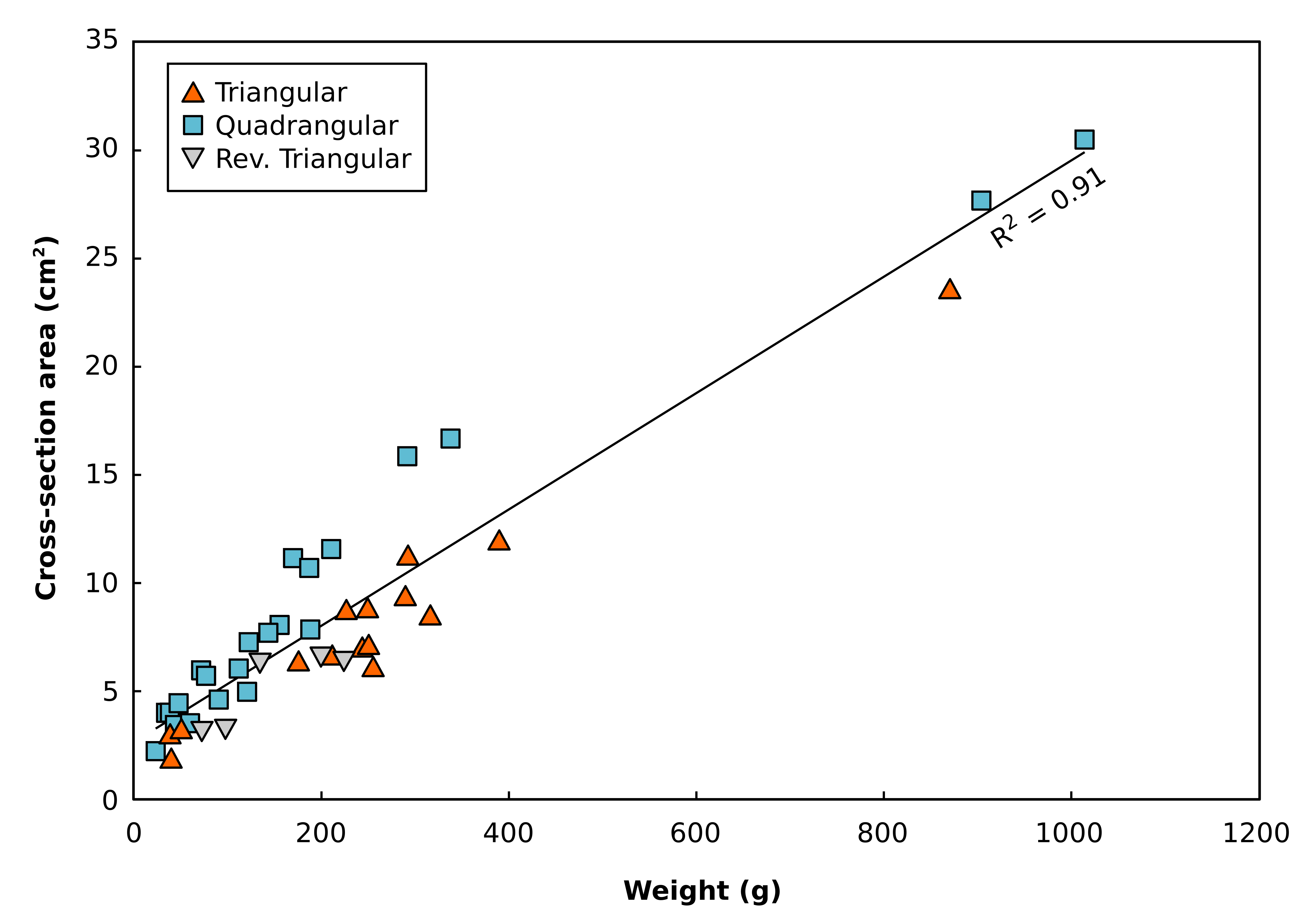

Supplement: S2 Fig — The regression line is for all cross-section shapes combined. Data are from [49]. (TIFF) [file pone.0188207.s004.tiff]
