# Supplementary material for: Basalt geochemistry reveals high frequency of prehistoric tool exchange in low hierarchy Marquesas Islands (Polynesia)
Source: PLoS One. 2017 Dec 27;12(12):e0188207. doi: 10.1371/journal.pone.0188207 (PMC5744946; doi:10.1371/journal.pone.0188207)
Supplement: S1 Table — (DOCX) [file pone.0188207.s005.docx]

**S1a Table.** Summary data for first discriminant function analysis.

|  |  | | |  | |  | | | |  | | |  | | |  | |  | |  | | | |  | |  |
| --- | --- | --- | --- | --- | --- | --- | --- | --- | --- | --- | --- | --- | --- | --- | --- | --- | --- | --- | --- | --- | --- | --- | --- | --- | --- | --- |
| **Pooled Within-Groups Correlation Matrix** | | | | | | | | | | | | | | | | | | | | | | | | | | |
|  | **TiO2** | | **Mn** | | **Fe2O3** | | | | **Ni** | | | **Cu** | | | **Zn** | | | | **Rb** | | | **Sr** | | | **Nb** | |
| CaO | 0.28 | | 0.23 | | -0.02 | | | | 0.14 | | | 0.01 | | | -0.23 | | | | -0.08 | | | 0.06 | | | -0.10 | |
| TiO2 |  | | 0.15 | | 0.10 | | | | 0.15 | | | -0.04 | | | -0.12 | | | | -0.04 | | | 0.18 | | | 0.08 | |
| Mn |  | |  | | 0.43 | | | | 0.36 | | | 0.12 | | | 0.11 | | | | 0.02 | | | -0.03 | | | -0.13 | |
| Fe2O3 |  | |  | |  | | | | 0.02 | | | 0.13 | | | 0.60 | | | | 0.09 | | | 0.10 | | | -0.04 | |
| Ni |  | |  | |  | | | |  | | | 0.23 | | | -0.17 | | | | 0.19 | | | -0.07 | | | 0.10 | |
| Cu |  | |  | |  | | | |  | | |  | | | 0.06 | | | | 0.27 | | | 0.07 | | | 0.18 | |
| Zn |  | |  | |  | | | |  | | |  | | |  | | | | 0.05 | | | 0.03 | | | -0.06 | |
| Rb |  | |  | |  | | | |  | | |  | | |  | | | |  | | | 0.30 | | | 0.50 | |
| Sr |  | |  | |  | | | |  | | |  | | |  | | | |  | | |  | | | 0.47 | |
|  |  | | |  | |  | | | |  | | |  | | |  | |  | |  | | | |  | |  |
|  |  | | |  | |  | | | |  | | |  | | |  | |  | |  | | | |  | |  |
| **Standardized Canonical Discriminant Function Coefficients** | | | | | | | | | | | | | | | | | | | | | | | | | | |
|  | **CaO** | | | **TiO2** | | **Mn** | | | | **Fe2O3** | | | **Ni** | | | **Cu** | | **Zn** | | **Rb** | | | | **Sr** | | **Nb** |
| Function 1 | 0.52 | | | 0.07 | | -0.37 | | | | 0.21 | | | 0.07 | | | 0.50 | | 0.02 | | -0.23 | | | | -0.58 | | -0.18 |
| Function 2 | 0.28 | | | -0.42 | | 0.71 | | | | -0.65 | | | -0.26 | | | 0.35 | | 0.32 | | 0.06 | | | | -0.28 | | 0.62 |
|  |  | | |  | |  | | | |  | | |  | | |  | |  | |  | | | |  | |  |
|  |  | | |  | |  | | | |  | | |  | | |  | |  | |  | | | |  | |  |
| **Eigenvalues** | | | | | | | | | | | | | | | | | | | | | | | | | | |
|  | **Eigenvalue** | | | | | | **% of Variance** | | | | | | | **Cumulative %** | | | | | | | **Canonical Correlation** | | | | | |
| Function 1 | 9.592 | | | | | | 70.9 | | | | | | | 70.9 | | | | | | | 0.952 | | | | | |
| Function 2 | 3.931 | | | | | | 29.1 | | | | | | | 100.0 | | | | | | | 0.893 | | | | | |
|  |  | | |  | |  | | | |  | | |  | | |  | |  | |  | | | |  | |  |
|  |  | | |  | |  | | | |  | | |  | | |  | |  | |  | | | |  | |  |
| **Classification Results** | | | | | | | | | | | | | | | | | | | | | | | | | | |
|  | |  | | | | | | **Predicted Group** | | | | | | | | | | | | | | |  | | | |
|  | |  | | | | | | **Group A** | | | **Group B** | | | | | | **Group C** | | | | | | **Total** | | | |
| Actual | | Group A | | | | | | 201 | | |  | | | | | |  | | | | | | 201 | | | |
| Group | | Group B | | | | | |  | | | 244 | | | | | |  | | | | | | 244 | | | |
|  | | Group C | | | | | |  | | |  | | | | | | 52 | | | | | | 52 | | | |
|  | | **Artifacts** | | | | | | **132** | | | **141** | | | | | | **3** | | | | | | **276** | | | |
|  | |  | | | | | |  | | |  | | | | | |  | | | | | |  | | | |
| Cross-validated | | Group A | | | | | | 201 | | |  | | | | | |  | | | | | | 201 | | | |
|  | | Group B | | | | | | 1 | | | 243 | | | | | |  | | | | | | 244 | | | |
|  | | Group C | | | | | |  | | |  | | | | | | 52 | | | | | | 52 | | | |
| 100.0% of original grouped cases correctly classified. | | | | | | | | | | | | | | | | | | | | | | | | | | |
| 99.8% of cross-validated grouped cases correctly classified. | | | | | | | | | | | | | | | | | | | | | | | | | | |

**S1ba Table.** Summary data for Discriminant Function Analysis of Group A.

|  |  | | |  | | |  | | | | |  | | |  | | |  | |  | | |  | | |  | | |  |
| --- | --- | --- | --- | --- | --- | --- | --- | --- | --- | --- | --- | --- | --- | --- | --- | --- | --- | --- | --- | --- | --- | --- | --- | --- | --- | --- | --- | --- | --- |
| **Pooled Within-Groups Correlation Matrix** | | | | | | | | | | | | | | | | | | | | | | | | | | | | | |
|  | **Cu** | | | | | **Zn** | | | | | | | **Rb** | | | | | | **Sr** | | | | | | **Zr** | | | | |
| Ni | 0.17 | | | | | -0.07 | | | | | | | -0.14 | | | | | | -0.37 | | | | | | -0.17 | | | | |
| Cu |  | | | | | -0.27 | | | | | | | 0.28 | | | | | | 0.08 | | | | | | 0.43 | | | | |
| Zn |  | | | | |  | | | | | | | -0.12 | | | | | | -0.04 | | | | | | -0.11 | | | | |
| Rb |  | | | | |  | | | | | | |  | | | | | | 0.39 | | | | | | 0.44 | | | | |
| Sr |  | | | | |  | | | | | | |  | | | | | |  | | | | | | 0.68 | | | | |
|  |  | | |  | | |  | | | | |  | | |  | | |  | |  | | |  | | |  | | |  |
|  |  | | |  | | |  | | | | |  | | |  | | |  | |  | | |  | | |  | | |  |
| **Standardized Canonical Discriminant Function Coefficients** | | | | | | | | | | | | | | | | | | | | | | | | | | | | | |
|  | **Ni** | | | | **Cu** | | | | | | **Zn** | | | | | | **Rb** | | | | **Sr** | | | | | | **Zr** | | |
| Function 1 | 0.90 | | | | -0.29 | | | | | | 0.28 | | | | | | 0.10 | | | | 0.64 | | | | | | 0.26 | | |
| Function 2 | -0.54 | | | | 0.20 | | | | | | 0.27 | | | | | | -0.54 | | | | 0.16 | | | | | | 0.65 | | |
|  |  | | |  | | |  | | | | |  | | |  | | |  | |  | | |  | | |  | | |  |
|  |  | | |  | | |  | | | | |  | | |  | | |  | |  | | |  | | |  | | |  |
| **Eigenvalues** | | | | | | | | | | | | | | | | | | | | | | | | | | | | | |
|  | **Eigenvalue** | | | | | | | **% of Variance** | | | | | | | | **Cumulative %** | | | | | | | | **Canonical Correlation** | | | | | |
| Function 1 | 3.254 | | | | | | | 64.0 | | | | | | | | 64.0 | | | | | | | | 0.875 | | | | | |
| Function 2 | 1.828 | | | | | | | 36.0 | | | | | | | | 100.0 | | | | | | | | 0.804 | | | | | |
|  |  | | |  | | |  | | | | |  | | |  | | |  | |  | | |  | | |  | | |  |
|  |  | | |  | | |  | | | | |  | | |  | | |  | |  | | |  | | |  | | |  |
| **Classification Results** | | | | | | | | | | | | | | | | | | | | | | | | | | | | | |
|  | | |  | | | | | | **Predicted Source** | | | | | | | | | | | | | | | | | | |  | |
|  | | |  | | | | | | **Northeast NH** | | | | | **Northwest NH** | | | | | | | | **Henua Ataha NH** | | | | | | **Total** | |
| Actual | | Northeast NH | | | | | | | | 157 | | | | 1 | | | | | | | |  | | | | | | 158 | |
| Source | | Northwest NH | | | | | | | | 1 | | | | 30 | | | | | | | |  | | | | | | 31 | |
|  | | Henua Ataha NH | | | | | | | |  | | | |  | | | | | | | | 12 | | | | | | 12 | |
|  | | **Artifacts** | | | | | | | | **95** | | | | **11** | | | | | | | | **26** | | | | | | **132** | |
|  | |  | | | | | | | |  | | | |  | | | | | | | |  | | | | | |  | |
| Cross-validated | | Northeast NH | | | | | | | | 156 | | | | 2 | | | | | | | |  | | | | | | 158 | |
|  | | Northwest NH | | | | | | | | 1 | | | | 30 | | | | | | | |  | | | | | | 31 | |
|  | | Henua Ataha NH | | | | | | | |  | | | |  | | | | | | | | 12 | | | | | | 12 | |
| 99.0% of original grouped cases correctly classified. | | | | | | | | | | | | | | | | | | | | | | | | | | | | | |
| 98.5% of cross-validated grouped cases correctly classified. | | | | | | | | | | | | | | | | | | | | | | | | | | | | | |

**S1c Table.** Summary data for discriminant function analysis of Group B.

|  |  | | |  | | |  | | | | | |  | |  | | | | |  | |  | | |  | | | |  | | |  |  |
| --- | --- | --- | --- | --- | --- | --- | --- | --- | --- | --- | --- | --- | --- | --- | --- | --- | --- | --- | --- | --- | --- | --- | --- | --- | --- | --- | --- | --- | --- | --- | --- | --- | --- |
| **Pooled Within-Groups Correlation Matrix** | | | | | | | | | | | | | | | | | | | | | | | | | | | | | | | | |  |
|  | **Ni** | | | | | **Cu** | | | | | | **Zn** | | | | | | **Rb** | | | | | **Sr** | | | | | | | **Nb** | | | |
| Fe_2_O_3_ | -0.17 | | | | | 0.28 | | | | | | 0.77 | | | | | | 0.13 | | | | | -0.08 | | | | | | | -0.15 | | | |
| Ni |  | | | | | 0.03 | | | | | | -0.35 | | | | | | -0.24 | | | | | -0.07 | | | | | | | 0.05 | | | |
| Cu |  | | | | |  | | | | | | 0.28 | | | | | | 0.08 | | | | | -0.03 | | | | | | | 0.05 | | | |
| Zn |  | | | | |  | | | | | |  | | | | | | 0.27 | | | | | 0.02 | | | | | | | -0.12 | | | |
| Rb |  | | | | |  | | | | | |  | | | | | |  | | | | | 0.18 | | | | | | | 0.1 | | | |
| Sr |  | | | | |  | | | | | |  | | | | | |  | | | | |  | | | | | | | 0.15 | | | |
|  |  | | |  | | |  | | | | | |  | |  | | | | |  | |  | | |  | | | |  | | |  |  |
|  |  | | |  | | |  | | | | | |  | |  | | | | |  | |  | | |  | | | |  | | |  |  |
| **Standardized Canonical Discriminant Function Coefficients** | | | | | | | | | | | | | | | | | | | | | | | | | | | | | | | | |  |
|  | **Fe_2_O_3_** | | | | **Ni** | | | | | | **Cu** | | | **Zn** | | | | | | | **Rb** | | | **Sr** | | | | | | | **Nb** | | |
| Function 1 | -0.44 | | | | 0.62 | | | | | | 0.31 | | | 0.34 | | | | | | | 0.69 | | | -0.30 | | | | | | | 0.27 | | |
|  |  | | |  | | |  | | | | | |  | |  | | | | |  | |  | | |  | | | |  | | |  |  |
|  |  | | |  | | |  | | | | | |  | |  | | | | |  | |  | | |  | | | |  | | |  |  |
| **Eigenvalues** | | | | | | | | | | | | | | | | | | | | | | | | | | | | | | | | |  |
|  | **Eigenvalue** | | | | | | | **% of Variance** | | | | | | | | **Cumulative %** | | | | | | | | | | **Canonical Correlation** | | | | | | | |
| Function 1 | 3.288 | | | | | | | 100.0 | | | | | | | | 100.0 | | | | | | | | | | 0.876 | | | | | | | |
|  |  | | |  | | |  | | | | | |  | |  | | | | |  | |  | | |  | | | |  | | |  |  |
|  |  | | |  | | |  | | | | | |  | |  | | | | |  | |  | | |  | | | |  | | |  |  |
| **Classification Results** | | | | | | | | | | | | | | | | | | | | | | | | | | | | | | | | | |
|  | | |  | | | | | | **Predicted Source** | | | | | | | | | | | | | | | | | |  | | | | | | |
|  | | |  | | | | | | **Eiao Group 1** | | | | | | | | **Atikea NH** | | | | | | | | | | **Total** | | | | | | |
| Actual | | Eiao Group 1 | | | | | | | | 209 | | | | | | | | | 2 | | | | | | | | | 211 | | | | | |
| Source | | Atikea NH | | | | | | | |  | | | | | | | | | 33 | | | | | | | | | 33 | | | | | |
|  | | **Artifacts** | | | | | | | | **139** | | | | | | | | | **2** | | | | | | | | | **141** | | | | | |
|  | |  | | | | | | | |  | | | | | | | | |  | | | | | | | | |  | | | | | |
| Cross-validated | | Eiao Group 1 | | | | | | | | 209 | | | | | | | | | 2 | | | | | | | | | 211 | | | | | |
|  | | Atikea NH | | | | | | | | 1 | | | | | | | | | 32 | | | | | | | | | 33 | | | | | |
| 99.2% of original grouped cases correctly classified. | | | | | | | | | | | | | | | | | | | | | | | | | | | | | | | | | |
| 98.8% of cross-validated grouped cases correctly classified. | | | | | | | | | | | | | | | | | | | | | | | | | | | | | | | | | |

**S1d Table.** Summary data for discriminant function analysis of Group C.

|  |  | | |  | | |  | | | | |  | | |  | | |  | |  | | |  | | |  | | |  |
| --- | --- | --- | --- | --- | --- | --- | --- | --- | --- | --- | --- | --- | --- | --- | --- | --- | --- | --- | --- | --- | --- | --- | --- | --- | --- | --- | --- | --- | --- |
| **Pooled Within-Groups Correlation Matrix** | | | | | | | | | | | | | | | | | | | | | | | | | | | | | |
|  | **Fe_2_O_3_** | | | | | **Cu** | | | | | | | **Rb** | | | | | | **Sr** | | | | | | **Zr** | | | | |
| Mn | 0.47 | | | | | 0.29 | | | | | | | -0.28 | | | | | | -0.23 | | | | | | -0.03 | | | | |
| Fe_2_O_3_ |  | | | | | -0.21 | | | | | | | -0.26 | | | | | | -0.31 | | | | | | 0.15 | | | | |
| Cu |  | | | | |  | | | | | | | -0.46 | | | | | | -0.17 | | | | | | -0.49 | | | | |
| Rb |  | | | | |  | | | | | | |  | | | | | | 0.31 | | | | | | 0.6 | | | | |
| Sr |  | | | | |  | | | | | | |  | | | | | |  | | | | | | 0.4 | | | | |
|  |  | | |  | | |  | | | | |  | | |  | | |  | |  | | |  | | |  | | |  |
|  |  | | |  | | |  | | | | |  | | |  | | |  | |  | | |  | | |  | | |  |
| **Standardized Canonical Discriminant Function Coefficients** | | | | | | | | | | | | | | | | | | | | | | | | | | | | | |
|  | **Mn** | | | | **Fe_2_O_3_** | | | | | | **Cu** | | | | | | **Rb** | | | | **Sr** | | | | | | **Zr** | | |
| Function 1 | -0.22 | | | | 1.09 | | | | | | 0.65 | | | | | | 1.13 | | | | 0.566 | | | | | | -1.143 | | |
| Function 2 | 0.57 | | | | 0.26 | | | | | | -0.83 | | | | | | -0.06 | | | | 0.612 | | | | | | -0.302 | | |
|  |  | | |  | | |  | | | | |  | | |  | | |  | |  | | |  | | |  | | |  |
|  |  | | |  | | |  | | | | |  | | |  | | |  | |  | | |  | | |  | | |  |
| **Eigenvalues** | | | | | | | | | | | | | | | | | | | | | | | | | | | | | |
|  | **Eigenvalue** | | | | | | | **% of Variance** | | | | | | | | **Cumulative %** | | | | | | | | **Canonical Correlation** | | | | | |
| Function 1 | 42.699 | | | | | | | 96.2 | | | | | | | | 96.2 | | | | | | | | 0.988 | | | | | |
| Function 2 | 1.695 | | | | | | | 3.8 | | | | | | | | 100.0 | | | | | | | | 0.793 | | | | | |
|  |  | | |  | | |  | | | | |  | | |  | | |  | |  | | |  | | |  | | |  |
|  |  | | |  | | |  | | | | |  | | |  | | |  | |  | | |  | | |  | | |  |
| **Classification Results** | | | | | | | | | | | | | | | | | | | | | | | | | | | | | |
|  | | |  | | | | | | **Predicted source** | | | | | | | | | | | | | | | | | | |  | |
|  | | |  | | | | | | **Hiva Oa** | | | | | **Taiohae NH** | | | | | | | | **Eiao Group II** | | | | | | **Total** | |
| Actual | | Hiva Oa | | | | | | | | 6 | | | |  | | | | | | | |  | | | | | | 6 | |
| Source | | Taiohae NH | | | | | | | |  | | | | 23 | | | | | | | |  | | | | | | 23 | |
|  | | Eiao Group II | | | | | | | |  | | | |  | | | | | | | | 23 | | | | | | 23 | |
|  | | **Artifacts** | | | | | | | |  | | | | **3** | | | | | | | |  | | | | | | **3** | |
|  | |  | | | | | | | |  | | | |  | | | | | | | |  | | | | | |  | |
| Cross-validated | | Hiva Oa | | | | | | | | 6 | | | |  | | | | | | | |  | | | | | | 6 | |
|  | | Taiohae NH | | | | | | | |  | | | | 23 | | | | | | | |  | | | | | | 23 | |
|  | | Eiao Group II | | | | | | | |  | | | |  | | | | | | | | 23 | | | | | | 23 | |
| 100.0% of original grouped cases correctly classified. | | | | | | | | | | | | | | | | | | | | | | | | | | | | | |
| 100.0% of cross-validated grouped cases correctly classified. | | | | | | | | | | | | | | | | | | | | | | | | | | | | | |
