# Supplementary material for: Basalt geochemistry reveals high frequency of prehistoric tool exchange in low hierarchy Marquesas Islands (Polynesia)
Source: PLoS One. 2017 Dec 27;12(12):e0188207. doi: 10.1371/journal.pone.0188207 (PMC5744946; doi:10.1371/journal.pone.0188207)
Supplement: S2 Table — (DOCX) [file pone.0188207.s006.docx]

**S2 Table.** Summary statistics for correlations between weight (g) and shoulder cross-section area (cm^2^) for complete adzes and preforms.

| **Form** | ***n*** | **R^2^** | ***F*-score** | ***t*-score** | ***P*-value** |
| --- | --- | --- | --- | --- | --- |
| Triangular | 14 | 0.954 | 257.49 | 16.05 | <0.001 |
| Rev. Triangular | 5 | 0.756 | 9.29 | 3.05 | 0.056 |
| Quadrangular | 21 | 0.935 | 162.11 | 12.73 | <0.001 |
|  |  |  |  |  |  |
| Preform | 28 | 0.905 | 285.04 | 16.88 | <0.001 |
| Adze | 14 | 0.897 | 104.30 | 10.21 | <0.001 |
|  |  |  |  |  |  |
| Combined | 42 | 0.909 | 388.35 | 19.71 | <0.001 |
