# Supplementary material for: Basalt geochemistry reveals high frequency of prehistoric tool exchange in low hierarchy Marquesas Islands (Polynesia)
Source: PLoS One. 2017 Dec 27;12(12):e0188207. doi: 10.1371/journal.pone.0188207 (PMC5744946; doi:10.1371/journal.pone.0188207)
Supplement: S3 Table — (DOCX) [file pone.0188207.s007.docx]

**S3 Table.** Comparison of absolute and relative bevel width by cross-section shape and source island.

| **Cross-section** | **Assigned**  **Source Island** | ***n*** | **Mean**  **(mm)** | **St.dev** | **Min**  **(mm)** | **Max.**  **(mm)** | ***t*-score** | **df** | ***P*-value** |
| --- | --- | --- | --- | --- | --- | --- | --- | --- | --- |
| *Absolute bevel width* | | | | | | | | | |
| Triangular | Eiao | 14 | 18.8 | 8.1 | 9 | 32 | 0.938 | 37 | 0.355 |
|  | Nuku Hiva | 25 | 21.5 | 8.8 | 10 | 51 |  |  |  |
|  |  |  |  |  |  |  |  |  |  |
| Quadrangular | Eiao | 27 | 33.3 | 17.6 | 7 | 91 | 0.304 | 41 | 0.763 |
|  | Nuku Hiva | 16 | 34.7 | 8.3 | 24 | 54 |  |  |  |
|  |  |  |  |  |  |  |  |  |  |
| Rev. Triangular | Eiao | 5 | 32.0 | 9.7 | 25 | 48 | 2.189 | 11 | 0.051 |
|  | Nuku Hiva | 8 | 42.5 | 7.5 | 30 | 50 |  |  |  |
|  |  |  |  |  |  |  |  |  |  |
| Combined | Eiao | 46 | 29.0 | 15.7 | 7 | 91 | 0.077 | 93 | 0.939 |
|  | Nuku Hiva | 49 | 29.3 | 11.8 | 10 | 54 |  |  |  |
| *Relative bevel width* | | | | | | | | | |
| Triangular | Eiao | 14 | 0.58 | 0.20 | 0.32 | 1.01 | 0.055 | 37 | 0.956 |
|  | Nuku Hiva | 25 | 0.58 | 0.17 | 0.33 | 1.11 |  |  |  |
|  |  |  |  |  |  |  |  |  |  |
| Quadrangular | Eiao | 27 | 0.97 | 0.20 | 0.34 | 1.32 | 0.176 | 41 | 0.861 |
|  | Nuku Hiva | 16 | 0.98 | 0.15 | 0.75 | 1.24 |  |  |  |
|  |  |  |  |  |  |  |  |  |  |
| Rev. Triangular | Eiao | 5 | 0.99 | 0.17 | 0.77 | 1.17 | 0.806 | 11 | 0.437 |
|  | Nuku Hiva | 8 | 1.05 | 0.10 | 0.90 | 1.17 |  |  |  |
|  |  |  |  |  |  |  |  |  |  |
| Combined | Eiao | 46 | 0.85 | 0.27 | 0.32 | 1.32 | 1.175 | 93 | 0.243 |
|  | Nuku Hiva | 49 | 0.79 | 0.26 | 0.33 | 1.24 |  |  |  |
