# Supplementary material for: Basalt geochemistry reveals high frequency of prehistoric tool exchange in low hierarchy Marquesas Islands (Polynesia)
Source: PLoS One. 2017 Dec 27;12(12):e0188207. doi: 10.1371/journal.pone.0188207 (PMC5744946; doi:10.1371/journal.pone.0188207)
Supplement: S4 Table — Finished adzes were identified by the presence of polish on one or more surface. (DOCX) [file pone.0188207.s008.docx]

**S4 Table.** Frequencies of artifacts sourced to Eiao Island by manufacturing stage. Finished adzes were identified by the presence of polish on one or more surface.

|  | **Valley** | | | |  |
| --- | --- | --- | --- | --- | --- |
| **Manufacturing**  **Stage** | **Pua** | **Hakaea** | **Hatiheu** | **Anaho** | **Total** |
|  |  |  |  |  |  |
| *Count* |  |  |  |  |  |
| Finished | 9 | 28 | 14 | 28 | 79 |
| Unfinished | 13 | 15 | 8 | 16 | 52 |
| Indeterminate |  |  | 1 | 7 | 8 |
| **Total** | **22** | **43** | **23** | **51** | **139** |
|  |  |  |  |  |  |
| *Percent* |  |  |  |  |  |
| Finished | 40.9 | 65.1 | 60.9 | 54.9 | 56.8 |
| Unfinished | 59.1 | 34.9 | 34.8 | 31.4 | 37.4 |
| Indeterminate |  |  | 4.3 | 13.7 | 5.8 |
